# Genetic Variants at the Nebulette Locus Are Associated with Myxomatous Mitral Valve Disease Severity in Cavalier King Charles Spaniels

**DOI:** 10.3390/genes13122292

**Published:** 2022-12-05

**Authors:** Sophie E. Mead, Niek J. Beijerink, Mitchell O’Brien, Claire M. Wade

**Affiliations:** 1 Faculty of Science, School of Life and Environmental Sciences Camperdown, The University of Sydney, Sydney, NSW 2006, Australia; 2 Veterinaire Specialisten Vught, Reutsedijk 8a, 5264 Vught, The Netherlands; 3 Transformational Bioinformatics, Health and Biosecurity, Commonwealth Scientific and Industrial Research Organisation (CSIRO), Westmead, NSW 2145, Australia

**Keywords:** congestive heart failure, dog, genetics, heart disease, myxomatous mitral valve disease, nebulette

## Abstract

The most common cardiovascular disease in domestic dogs is myxomatous mitral valve disease (MMVD), accounting for 75% of all cardiac disease. An increase in age is generally associated with increased incidence of the disease, but Cavalier King Charles Spaniels (CKCS) exhibit an unusually high prevalence of early-onset MMVD, and thus, potentially greater cardiac morbidity and mortality compared to other breeds. Previous research has suggested that selected candidate risk alleles for MMVD are fixed in CKCSs, including six locations within the *Nebulette* (*NEBL*) gene on CFA2. The current study analysed genotypes of 180 Australian CKCSs at the identified risk loci. Of these, 178 were phenotyped for severity of disease by echocardiographic measurements of left atrium to aortic root ratio (LA:Ao) and weight normalised left ventricular end diastolic diameter (LVIDdN). Genotyping array markers correctly predicted the genotype at the risk-variant loci in the CKCS population, and the *NEBL1*, *NEBL2* and *NEBL3* variants were observed to be in perfect linkage disequilibrium in this cohort. The CKCS cohort included 6/178 dogs being heterozygous for the protective/wild-type alleles at the NEBL locus. The mean LA:Ao and LVIDdN scores of these dogs heterozygous at *NEBL1-3* variants were significantly smaller, and with significantly lower variance compared to age-matched CKCSs that were homozygous for risk alleles. The lower cardiac measurements in the heterozygous dogs indicate a significantly reduced risk of severe MMVD disease. Our analysis suggests that despite relative fixation of the NEBL risk alleles, healthy reference alleles at *NEBL1-3* exist in low frequency in the CKCS breed and can be used to reduce MMVD severity and mortality.

## 1. Introduction

Myxomatous mitral valve disease (MMVD) (OMIA—000654-9615) is a disease affecting the mitral valve of domestic dogs (*Canis lupus familiaris*). MMVD is characterised by slow progressive degenerative mitral valve changes, resulting in mitral valve prolapse and regurgitation (MR) and subsequent left atrial (LA) and ventricular (LV) dilatation [1]. Long-standing LV and LA volume overload will, in affected dogs, ultimately progress to congestive heart failure (CHF) when fluid begins to accumulate in the lungs [2]. Although the process of the degenerative valve remodelling has been well described, the underlying causes and mechanisms involved in the development and progression of the disease remain poorly characterized [3].

One widely used staging system to describe how dogs with MMVD might progress divides affected dogs into four categories. Those at risk for developing the disease are considered to be at stage A; those with evidence of MR and no signs of CHF are at stage B; those with signs of CHF are at stage C; and those with signs of CHF refractory to treatment are considered to be at stage D. Dogs with early stage B disease and no evidence of cardiac enlargement are categorized as stage B1; dogs in which cardiac enlargement has developed in order to compensate for the volume load, but which have not yet developed signs of CHF, are categorized as stage B2 [4]. Part of the criteria for distinguishing MMVD stage is the echocardiographic assessment of LA to aortic root ratio (LA:Ao) and weight normalised LV internal diameter in diastole (LVIDdN), with these dimensions roughly increasing proportionally with severity of disease [4].

MMVD accounts for 75% of all cardiac disease in domestic dogs, and the CKCS exhibits an unusually high prevalence of early-onset and severe MMVD [5]. Prevalence and incidence are both related to the breed and age of the animal [6]. Smaller breeds are generally at higher risk of MMVD, with particularly high incidences in Cavalier King Charles Spaniels (CKCS) [4,7,8]. An increase in age in dogs also sees a significant increase in MMVD disease incidence in CKCSs, with a total 85% of dogs affected by 13 years of age [7,9]. Further, CKCSs have been observed to exhibit an unusually high prevalence of early-onset MMVD [8,10]. Expressivity of this condition is variable, with some dogs showing evidence of more severe disease at earlier ages than other dogs.

The CKCS is reported to be one of the most heavily bottlenecked domestic dog breeds as a result of multiple decades of closed population breeding [6,11]. As a result, CKCSs carry up to 13% more derived alleles than other domestic dog breeds [6], and this is believed to contribute to high-risk states for certain diseases, including MMVD. While the disease progression in CKCSs is not dissimilar to other dog breeds, the early onset of the disease in combination with rapid progression in some dogs leads to earlier mortalities and higher morbidity. Due to the known high morbidity and mortality in the CKCS, it is the most studied breed in relation to MMVD [12], yet the genetic basis of the disease is not well understood.

The suggestion of a genetic predisposition to MMVD existing within the CKCS breed was first introduced in 1996, with a study proposing that heart murmurs and MMVD were both polygenic threshold traits due to the exclusion of fully penetrant Mendelian inheritance models [13]. Following this, later research quantified heritability estimates for both the degree of a murmur (0.67 ± 0.07) and presence or absence of a murmur (0.33 ± 0.07) [7]. Transcriptomic studies have identified genes associated with cardiomyocytes that show gene fold changes and gene expression differences in the CKCS compared to other breeds, such as *Nebulette (NEBL)* [10]. This, however, does not definitively determine that these changes are involved in MMVD pathogenesis. Other researchers have utilised genome-wide association studies (GWAS) to pinpoint genes involved in MMVD pathogenesis though no gene variants have been verified by multiple researchers [9,14,15].

Most recently, a study featuring 20 United States and European CKCSs identified ten possible risk variants related to MMVD that are highly conserved in the breed [6]. Six of these variants were located in the *NEBL* gene, and the remaining four were located in the *Latrophilin 2 (LPHN2)*, *Sorbin and SH3 Domain Containing 2 (SORBS2)*, *5-Hydroxytryptamine Receptor 1F* (*HTR1F*) and *Hepatoma Derived Growth Factor-Like 1* (*HDGFL1)* genes. The study identified these variants via screening for evolutionarily conserved variants across mammals using the evolutionary divergence metric of pairwise F_st_. Researchers then identified potential MMVD-associated variants by comparing them with genes differentially expressed in a previous MMVD microarray [16]. The study concluded that these ten variants are evolutionary-conserved variants that presented at high frequency in the CKCS and were differentially expressed in the micro-array. Additionally, this study found that the genotype for one of the *NEBL* risk variants (*NEBL3*) was a significant predictor of graded MMVD disease status in the dachshund. Considering the previous finding of the gene expression differences in *NEBL* in the CKCS [10], this is concerning for the breed. If *NEBL* risk alleles are fixed in the CKCS breed and proven as causative for MMVD risk and/or severity, cross-breeding would be required to breed out severe MMVD in the CKCS.

This study seeks to investigate genotypes at the ten identified risk variant locations in an Australian cohort of CKCSs. The study will determine if the risk alleles are fixed in the wider CKCS population, and whether derived alleles at the variant sites are associated with MMVD disease severity as measured by LA:Ao and LVIDdN.

## 2. Materials and Methods

### 2.1. Blood Sampling and Echocardiographic Assessment

Blood samples from 183 privately-owned CKCSs in Australia were collected subject to the University of Sydney Animal Ethics Committee (Approval numbers: 2015/902 and 2018/1449). More severely affected dogs (those requiring treatment) were generally referred cases at the University Veterinary Teaching Hospital, Sydney. Asymptomatic dogs were generally recruited at ‘open sessions’ organised by local breed clubs. Each individual CKCS was auscultated and had an echocardiographic assessment by a board-certified veterinary cardiologist, with an MMVD grade assigned to each dog per the American College of Veterinary Internal Medicine’s (ACVIM) staging guidelines [4]. Dogs in stage A and B had to be at least 7 years of age, given the age-related penetrance of the disease, making it difficult to accurately phenotype an individual at an earlier age. Key heart measurements were taken during this assessment, including the sizes of the LA, aortic root and LV end diastolic diameter. These measurements were used to produce an LA to aortic root ratio (LA:Ao) and weight-normalised LV end diastolic diameter (LVIDdN) according to the following formulae: normalized LVIDd (LVIDdN) = LVIDd(cm)/(BW(kg))^0.294^. Both the LA:Ao and LVIDdN were then normalised for age using a regression-based adjustment that reported each animal’s LA:Ao and LVIDdN corrected to eight years of age. LA:Ao values were age-adjusted using the equation: Expected_LAAo for age = 0.038(AGE) + 1.2458, LaAoDeviation = ObservedLAAo − Expected_LAAo. From regression, AGE8_LaAO = 1.5498. LAAo corrected to 8 years = AGE8_LAAO + LaAoDeviation. The same equation was used to age-adjust all LVIDdN values.

### 2.2. DNA Extraction and Array-Based Genotyping

Genomic DNA was extracted from the whole-blood samples using the PureLink DNA Mini Kit (Invitrogen, Hilden, Germany) as per the manufacturer’s instructions. DNA quality was assessed using a NanoDrop Spectrophotometer (ThermoFisher Scientific, Waltham, MA, USA). DNA samples of 183 individuals were genotyped using the Illumina Canine 170K or 220K genotyping arrays (Neogen Inc., Lincoln, NE, USA).

### 2.3. Allele Frequencies for Risk Variants across Dogs

Wider CKCS breed frequencies for the candidate risk variants were assessed using public canine genotyping resource archives, comprising genome-wide variant calls from whole genome sequencing of 4 CKCSs included in the wider analysis of 590 canines [17], and genotyping-array based genotypes from a Canadian cohort of 96 CKCS [18].

### 2.4. Regional Analysis of Array Genotypes

Array genotypes within 500 kb of candidate risk variants identified by Axelsson et al. [10] (Table 1 and Table 2) were extracted from the genome-wide array genotypes using PLINK [19]. Minor allele frequencies for individual variants (--freq) and genotype counts (--freqx) were reported, with no quality filtering. The minor allele frequencies were tabulated and charted for each gene region, and marker genotypes were counted according to their genotyping status as homozygous-risk, homozygous wild-type or heterozygous. Risk gene regions demonstrating normal levels of polymorphism (MAF > 0.05) were noted as variable in the breed and not analysed further (Table 3).

### 2.5. Analysis of Risk Variants

PCR was performed on eight individuals in preparation for sequencing. The dogs chosen for sequencing were either heterozygous or homozygous for minor alleles at the chosen predictive array markers assigned to the presumptive risk variant locations. Only loci exhibiting mean minor allele frequencies less than 0.05 were sequenced at the target variant locations, as these showed strong fixation. *NEBL4*, *NEBL5* and *NEBL6* were excluded from sequencing as they are not likely to be significant in MMVD pathogenesis; *NEBL4* and *NEBL5* hold the reference alleles as the major allele in the CKCS and *NEBL6* was seen to be non-functional in previous work [6]. The *LPHN2*, *SORBS* and *HTR1F* variants were excluded from sequencing as they demonstrated normal levels of polymorphism (MAF > 0.10) in previous work [6] (Supplementary Table 18). PCR reactions were prepared using Amplitaq Gold PCR Mastermix (Applied Biosystems, Foster City, CA, USA) at a final volume of 20 µL using primers as described in Table 4.

Samples were denatured at 95 °C for 15 min, then underwent amplification for 35 cycles at 95 °C for 45 s, 57 °C (*NEBL*) or 58 °C (*HDGFL1*) for 45 s, 72 °C for 45 s, followed by elongation at 72 °C for 10 min. PCR products were visualised using gel electrophoresis with a 1.5% agarose gel. Three microlitres of SYBR green loading dye was added to 5 µL of PCR product and a 100 bp DNA ladder was utilised. The gel was run at 90 V for 45 min. To purify each sample, 15 µL of PCR product was dispensed into 6 µL of master mix containing 2 µL rSAP, 0.1 µL ExoI, 2 µL CutSmart Buffer and 1.9 µL water. Enzymatic activity was enabled for 30 min at 37 °C and then disabled for 15 min at 65 °C. The eight samples were sequenced using capillary separation on AB 3730 xl by the Australian Genome Research Facility (Westmead, Sydney, NSW, Australia). Concordance between variant genotype by sequencing and proposed array markers for the variant were assessed.

Gene regions demonstrating abnormal levels of polymorphism (MAF < 0.05) were analysed with a regional linkage disequilibrium (r2) to determine if array markers were concordant with variants using PLINK (--ld-window-r2) [19]. The *NEBL3* variant (included in the genotyping array) was analysed with a regional pairwise linkage disequilibrium to determine LD with other *NEBL* variants. The *HDGFL1* variant was analysed with a regional linkage disequilibrium to determine LD with the *HDGFL1* array marker.

### 2.6. Analysis of Physiological Data

Summary statistics for quantitative age-adjusted LA:Ao and LVIDdN heart phenotypes were compared according to a predicted risk variant genotype based on array markers in perfect concordance with risk variants. F-tests were performed to distinguish if variances in LA:Ao measurements between haplotype groups were significant using Microsoft Excel function *F-test Two-Sample for Variance* [20]. T-tests were performed to distinguish if means in LA:Ao measurements between haplotype groups were significant using Microsoft Excel function *t-Test: Two Sample Assuming Unequal Variances* [20]. The same analysis was performed for LVIDdN values. Age was analysed as a control variable and submitted to the same statistical analysis as LA:Ao and LVIDdN values.

## 3. Results

### 3.1. Phenotypic and Echocardiographic Measurements

Phenotypic and echocardiographic measurements were available for 178 dogs. The characteristics of the dogs are described in detail in Supplementary Table S1 and summarised in Table 5. The NEBL1-3 heterozygous cohort had a lower percentage of animals at stage B2 or above (17%) in comparison to the homozygous cohort (46%). The NEBL1-3 heterozygous cohort also had lower mean LA:Ao and LVIDdN values than their homozygous counterparts.

### 3.2. DNA Extraction and Array-Based Genotyping

Genotyping array data were available for 180 dogs, with three individuals lost due to poor quality. The final analysis cohort with matched genotype and phenotype data included 178 dogs. In all, 576 array variant sites were analysed for the five gene regions described by Axelsson et al. [6] with 91, 97, 153, 126 and 109 variants in each of the regions for *NEBL*, *LPHN2*, *HDGFL1*, *SORBS2* and *HTR1F*. Of the 576 markers included in the regional analyses, 45 were disregarded for poor genotype call rate, all within the *HTR1F* gene region. No markers were excluded for low minor allele frequency. Two of the array markers were exact variant locations, these being the *NEBL3* and *HTR1F* variants, all other variants were represented by the closest array marker in a 3′ direction. The array marker for *LPHN2* was the exception, as the closest 3′ marker was more than 8000 bp from the variant, and so the closest array marker in a 5′ direction was chosen.

### 3.3. Allele Frequencies for Risk Variants across Dog Breeds

Table 6 shows the reference allele frequencies from the global multi-breed 590 dogs [17], separated into three cohorts: all domestic dog breeds, just CKCS and just wolves. The Canadian [18] CKCS genotyping array markers for *NEBL3* are included directly, as these were equivalent to the variant location, and the *HDGFL1* variant genotype is inferred from linkage disequilibrium with nearby markers. The relative fixation of the derived allele in the CKCS compared with the other sampled dog breeds can be seen at all loci identified by Axelsson et al. [6], but particularly at the *NEBL1-3* (reference allele frequencies of 0.00–0.13), *NEBL6* (0.00) and *HDGFL1*(0.00) loci (Table 6).

### 3.4. Regional Analysis of Array Genotypes

**Figure 1:**
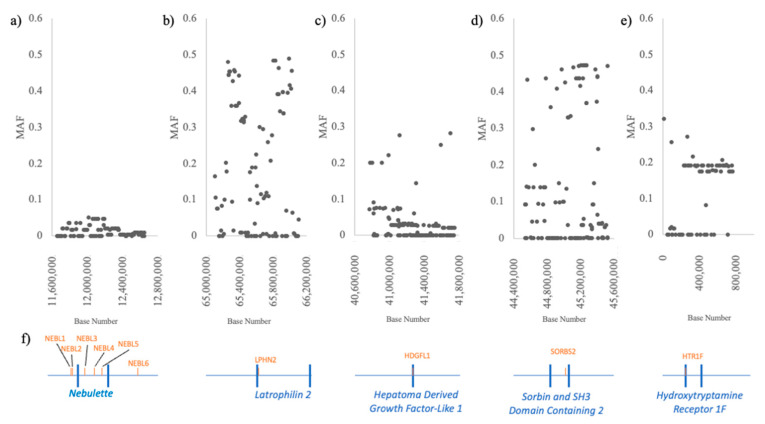
Minor allele frequency (MAF) of 180 genotyping array markers throughout the gene regions of (**a**) *NEBL* chr2:11,650,000 to chr2:12,650,000, (**b**) *LPHN2* chr6:65,109,000 to chr6:66,110,000, (**c**) *HDGFL1* chr7:40,745,000 to chr7:41,746,000, (**d**) *SORBS2* chr16:44,526,000 to chr16:45,527,000 and (**e**) *HTR1F* chr31:0 to chr31:773,000. (**f**) Gene regions marked across the x-axis, with variant locations marked above.

The positions, minor alleles and expected minor allele frequencies for each proposed risk variant can be seen in Table 1 and Table 2. The mean minor allele frequencies from our CKCS cohort across the gene regions can be seen in Table 1 and Table 2. Minor allele frequencies for each SNP are available in Supplementary Tables S2 and S3 and are plotted in Figure 1.

In the CKCS cohort, MAFs below 0.05 were considered as potentially fixed. Risk variants with MAF above 0.05 were regarded as polymorphic. The target risk genes with mean regional MAFs below 0.05 were investigated further, including variants from the proximal portion of *NEBL* including the region near *NEBL1*, *NEBL2* and *NEBL3* (mean MAF 0.01) and *HDGFL1* (mean MAF 0.03) (Table 3). 

### 3.5. Identified Disease-Risk Variants

Multiple dogs were found to be heterozygous at each of the risk loci. This included seven dogs at *HDGFL1*; six dogs at *NEBL1*, *NEBL2* and *NEBL3*; seventeen dogs at *NEBL4* and *NEBL5*; and three dogs at *NEBL6*. One dog was found to be homozygous for the reference allele at *HDGFL1*. The dogs heterozygous at the *NEBL* variant locations were otherwise not unusual and were within one standard deviation of the tested group mean.

Eight dogs harbouring reference alleles in the homozygous or heterozygous state at the *NEBL1-3* and *HDGFL1* variant locations were subjected to sequencing at AGRF. Five of the eight dogs were sequenced at the *NEBL1* and *NEBL2* variants, and all eight dogs were sequenced for the *NEBL3* and *HDGFL1* risk variants.

For all sequenced dogs, the tested risk variant genotype was perfectly concordant with the predictive array variant genotype. That is, dogs with heterozygous alleles at the array markers were also heterozygous at the risk variant locations. This included three dogs for the *HDGFL1*, *NEBL1* and *NEBL2* variants, and four dogs for the *NEBL3* variant (Table 1).

Sequencing identified additional variants within the sequenced fragments for *NEBL1* and *NEBL3*. Within the *NEBL1* fragment, variation was observed at position chr2:11,816,545 G > A (rs9112065) and this is an upstream variant (intronic in the NEBL isoform represented by NM_001377326 but 5′ to most other NEBL transcripts). The second new variant was found within the *NEBL3* fragment at position chr2:11,979,669 G > T (rs9212851) and is an intronic variant. These are both known variants, according to Ensembl [21]. Genotypes of sequenced dogs were universally concordant between the expected target risk variant allele and the newly identified variant allele.

All sequenced dogs that were heterozygous at *NEBL1* were also heterozygous at *NEBL2* and *NEBL3*.

**Figure 2:**
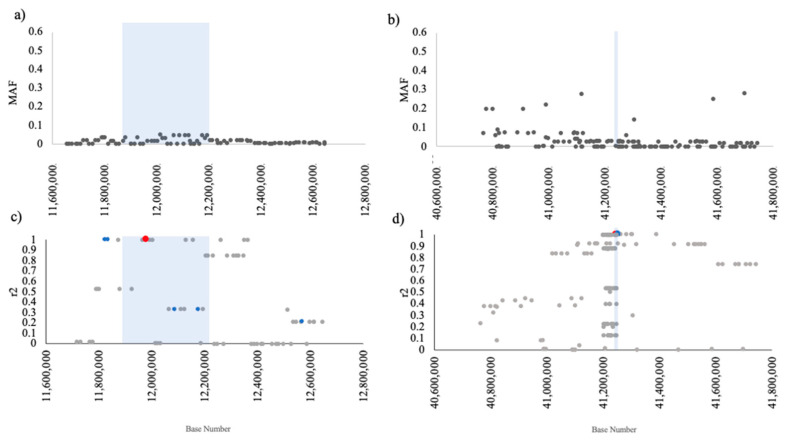
Characteristics of 180 Cavalier King Charles Spaniels (CKCS) at two low diversity loci as assayed using genotyping arrays. (**a**) Minor allele frequency (MAF) throughout the *NEBL* gene region chr2:11,650,000 to chr2:12,650,000, (**b**) MAF throughout the *HDGFL1* region chr7:40,745,000 to chr7:41,746,000. (**c**) Regional pairwise linkage disequilibrium by r2 (LD) (grey dots) with the *NEBL3* variant at chr2:11 979 724 (red dot) and other NEBL variant loci shown as blue dots. (**d**) Regional pairwise LD (grey dots) with the *HDGFL1* variant predictor chr7:41 248 384 (coding variant at 7:41 245 057) (blue dot). Shaded regions represent the respective gene spans.

The regional pairwise linkage disequilibrium between local array markers with *NEBL3* and *HDGFL1* can be seen in Figure 2c,d, respectively. Regional pairwise linkage disequilibrium showed that the array marker for *NEBL3* (which is equivalent to the variant location) is in perfect LD with array markers for *NEBL1* and *NEBL2*. Together, these variants are hereafter treated as a single haplotype.

Regional linkage disequilibrium showed imperfect LD (r2 = 0.53) between the *HDGFL1* variant and predictive array marker (chr7: 41 248 384; rs24433893) in CKCSs represented by the Jagannathan [17] cohort. The public data only included risk haplotype CKCSs with no heterozygous animals present.

### 3.6. Analysis of Physiological Data

The mean LA:Ao and LVIDdN heart metrics in the *NEBL1-5* homozygous dogs are significantly larger than the *NEBL1-5* heterozygous cohort. Animals that are heterozygous for the wild-type allele at *NEBL1-6* have mean LA:Ao and LVIDdN scores below the measurements required for a B2 stage (Table 7) and variances that exclude the B2 stage. Statistical tests quantitively showed significant differences between homozygous and heterozygous genotypes within the *NEBL1-5* variants’ heart measurements, with all *p*-values ≤0.01 for each variant (Table 7) for LVIDdN and LA:Ao.

The alleles at *NEBL6* and *HDGFL1* did not have a statistically significant effect on heart metrics as no significant difference was seen at either the *NEBL6* or *HDGFL1* variants between homozygous and heterozygous genotypes in the heart metrics (Table 7).

**Figure 3:**
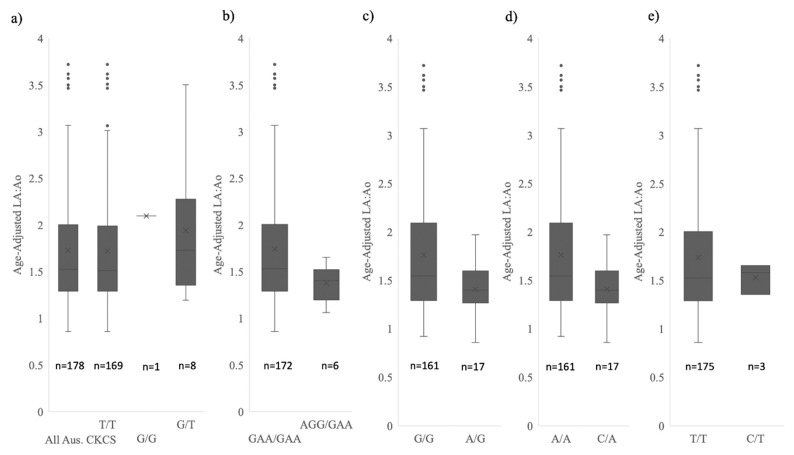
Comparison of age-adjusted LA:Ao measurements for 178 CKCSs at variant representative array markers for (**a**) *HDGFL1* (**b**) *NEBL1-3* (*NEBL1, NEBL2* and *NEBL3* grouped as they are in perfect LD, see Figure 2) (**c**) *NEBL4* (**d**) *NEBL5* and (**e**) *NEBL6*. Plots include median line, mean marker and quartile ranges. Alternate homozygote was only observed at *HDGFL1* predictive array marker.

**Figure 4:**
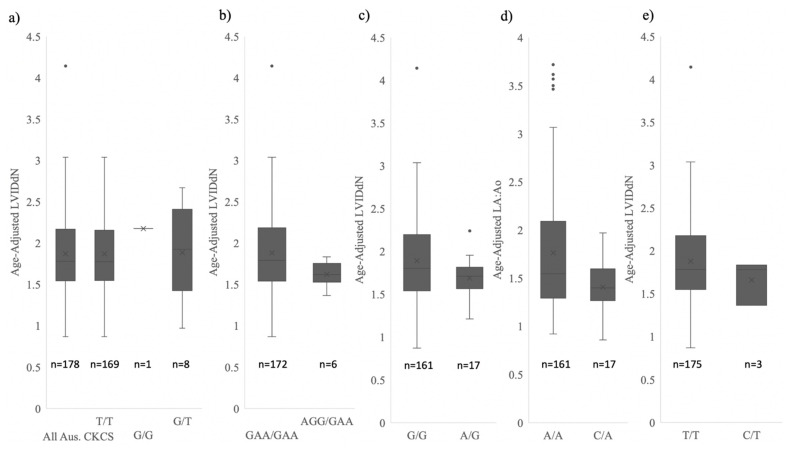
Comparison of age-adjusted LVIDdN measurements for 178 CKCSs at variant representative array markers for (**a**) *HDGFL1* (**b**) *NEBL1-3* (*NEBL1*, *NEBL2* and *NEBL3* grouped as they are in perfect LD, see Figure 2) (**c**) *NEBL4* (**d**) *NEBL5* and (**e**) *NEBL6*. Plots include median line, mean marker and quartile ranges. Alternate homozygote was only observed at *HDGFL1* predictive array marker.

Mean and variances for genotype classes at the *HDGFL1* and *NEBL1-6* risk variants can be visualised in Figure 3 and Figure 4. The variances in the LVIDdN and LA:Ao heart metrics in the heterozygous individuals for the *NEBL* variants are reduced compared to homozygous individuals. The range of LA:Ao and LVIDdN heart measurements within the *NEBL* heterozygous individuals excludes the ACVIM B2 stage [4]. This was confirmed with a means test which identified a significant difference between the B2 individuals and the *NEBL* heterozygous individuals (*p* < 0.01 for both LVIDdN and LA:Ao). Any heterozygous dogs at the *HDGFL1* variant are not physiologically dissimilar from the homozygous cohort.

## 4. Discussion

Our results find that variation exists at the previously identified MMVD risk loci [6], and that heterozygous animals at the *NEBL1-3* loci have smaller heart sizes, as indicated by LA:Ao and LVIDdN values.

Given the high minor allele frequencies observed within the *SORBS2*, *HTR1F* and *LPHN2* gene regions, the variants in those regions are likely unrelated to MMVD pathogenesis and further investigation of the variants did not proceed in this analysis. Axelsson et al. (2020) found that two of the variants (*NEBL4* and *NEBL5*) exhibited high frequencies of the reference alleles and were likely not relevant to MMVD risk in the breed [6]. The reference allele frequency for *NEBL1-3* and *HDGFL1* variants was 0.00 in the Axelsson et al. CKCS population [6]. This was determined by testing of the variant alleles directly in 20 individual CKCSs. In our Australian CKCS cohort, we identified multiple dogs to be heterozygous at the variant locations and nearby array markers. Genotype concordance with array prediction was confirmed for *NEBL* with perfect linkage disequilibrium between the *NEBL3* variant that is represented on the genotyping array with the *NEBL1* and *NEBL2* predictive array markers. We were unable to confirm LD between the *HDGFL1* variant and nearby predictive array markers because publicly available CKCSs with whole genome sequencing included only homozygous (risk haplotype) individuals. Our cohort was larger than the Axelsson et al. study [6], with 180 dogs compared to their 20, and our CKCS population includes a large proportion of older dogs enrolled in active heart screening. This may account for the increased wild-type allele frequency in our study population. When policy decisions prescribing population management interventions are envisaged, it is important to validate population characteristics in a larger representative cohort. Where intervention is indeed required, managed cross-breeding can be used to improve health status when risk variants are truly fixed.

When MMVD progresses, heart enlargement results in larger LVIDdN and LA:Ao measurements. Both LA:Ao and LVIDdN are standard measurements applied within the ACVIM MMVD staging guide [22]. The current analysis confirmed the assertion of Axelsson et al. [6] that individuals heterozygous at the variant locations of *NEBL1-3* exhibit significantly smaller LVIDdN and LA:Ao heart metrics. In agreement with Axelsson et al. [6], this study identifies that of the variants considered, *NEBL1-3* are the most physiologically significant. It is expected that the dogs heterozygous for wild-type alleles at these loci will experience reduced risk of mortality as a result of their lower LVIDdN and LA:Ao measurements.

Axelsson et al. [6] found that *NEBL1* and *NEBL2* variants were significantly expressed in cardiac papillary muscle, and that the *NEBL3* variant was a significant predictor of MMVD graded disease status in a dachshund population [6]. In agreement with our results, they found that *NEBL4-6* had no significant functional effect on heart health [6].

Mutations in the *nebulette* gene appear to be associated with heart enlargement, possibly due to a poorer quality cytoskeletal framework of cardiomyocytes [23,24,25]. Our results showing smaller heart measurements in dogs exhibiting the wild-type allele at the *NEBL* variant locations are expected due to the known function of *nebulette*. LA:Ao and LVIDdN are measurements of heart enlargement as a result of mitral regurgitation caused by MMVD. Dogs exhibiting protective alleles at the *NEBL* variant locations would be less likely to see enlarged heart chambers, as indicated by lower values of LA:Ao and LVIDdN in our CKCS cohort.

MMVD is complex in nature and unlikely to be controlled by a single gene. We expect that because of the CKCS breed history, some genes influencing this trait will have low variability. Other genes will be variable in the breed and lead to different individual risk levels within the CKCS population, with individual risk moderated by variants contributing to severity of disease. In this study, we focus on risk variants previously identified as relatively fixed in the breed by Axelsson et al. [6]. Once identified, the frequency of these favourable alleles can be increased if the right tools are made available to CKCS breeders. Since heterozygosity for the wild-type NEBL allele seems to reduce MMVD severity, CKCS breeders should plan to increase wild-type allele prevalence into the gene pool. There are two methods to accomplish this. The first is to apply genetic testing to identify registered CKCSs carrying the wild-type NEBL allele. Subsequently, breeders could be selecting for the wild-type allele, not against the derived allele, taking into consideration that selection for the wild-type allele should not reduce the gene pool too much. Second, breeders could introduce animals to the registry via an indexing scheme that enables the introduction of unregistered dogs (potentially of other breeds) carrying the wild-type NEBL allele into the registry system for this breed.

While crossbreeding is a viable option for improving heart health in the CKCS, rules across many canine registries currently preclude this approach and such rules will likely take a long time to change. Selection for favourable dominant alleles present in the breed can be applied immediately.

The *NEBL3* variant is already represented on the Illumina Canine High Density genotyping array, and variants NEBL1-3 are in perfect LD with each other and nearby array markers in the CKCS breed, opening the door for array marker panel-based testing for this breed.

This research demonstrates that healthy wild-type alleles exist within pedigreed CKCS at MMVD risk variant sites previously identified as fixed. The *NEBL1-3* variants are associated with MMVD risk with CKCS individuals heterozygous for wild-type alleles, demonstrating significantly improved LVIDdN and LA:Ao heart metrics. Our research shows that the severity of MMVD in the registered CKCS breed population can be reduced by genetic screening of dogs to identify those heterozygous for wild-type alleles at *NEBL1-3* that indicate lower risk of disease. Among CKCS, functionally significant variants of *NEBL1-3* are in perfect LD. Genetic screening using the *NEBL3* variant that is already present on the most commonly used genotyping array is expected to reduce the severity of MMVD in the pedigree-registered CKCS population.

## 5. Conclusions

This study has shown that the MMVD loci previously identified as fixed in the CKCS breed do exhibit low-level variation for the healthy reference alleles. Second, we have demonstrated that wild-type alleles at the *NEBL1*, *NEBL2* and *NEBL3* variant sites are significantly associated with improved metrics related to heart health. As many dogs included in this study were undergoing heart screening, this work provides some evidence that selection for improved heart health might increase incidence of desirable alleles at relevant risk loci. The *NEBL* gene is validated as important in cardiac health in CKCSs. Selection for the wild-type allele at *NEBL3* could decrease MMVD severity in the breed. As a low frequency variant in the CKCS breed, the NEBL3 wild-type allele may be difficult to capture and isolate for breeding purposes. It is also possible that crosses involving CKCSs beyond the first cross may be at increased risk of MMVD. Concentration for selection of this variant may increase the incidence of other health problems in the breed, and this study should be considered as a part of a holistic breeding program and not be used alone.

## Figures and Tables

**Table 1:** Genotyping results for eight Australian Cavalier King Charles Spaniels for four risk variants and predictive markers at the *NEBL1-3* & *HDGFL1* cardiac risk genes.

Gene Region	Variant	Consequence (Axelsson et al. [1] for Risk Variants)	Position (canFam3.1)	Reference Allele (canFam3)	Alternate Allele	Genotype							
						3256	3261	3262	3333	3334	3336	80271	80294
*Nebulette*	*NEBL1* risk variant	Regulation	chr2:11 816 535	A	G	A/G	G/G	G/G	A/G	A/G	G/G	A/G	A/G
*Nebulette*	New variant	Discovery	chr2:11 816 545	G	A	A/G	G/G	G/G	A/G	A/G	G/G	A/G	A/G
*Nebulette*	*NEBL1* predictive marker	Array marker	chr2:11 822 980	A	G	A/G	G/G	G/G	A/G	A/G	G/G	A/G	A/G
*Nebulette*	*NEBL2* risk variant	Regulation	chr2:11 823 576	C	T	-	-	-	C/T	C/T	-	C/T	C/T
*Nebulette*	*NEBL2* predictive marker	Array marker	chr2:11 832 538	G	A	G/A	A/A	A/A	G/A	G/A	A/A	G/A	G/A
*Nebulette*	New variant	Discovery	chr2:11 979 669	G	T	T/G	T/T	T/T	T/G	T/G	T/T	T/G	T/G
*Nebulette*	*NEBL3* risk variant (and predictive marker)	Candidate mutation	chr2:11 979 724	G	A	G/A	A/A	A/A	G/A	G/A	A/A	G/A	G/A
*Hepatoma Derived Growth Factor-Like 1*	*HDGFL1* risk variant	Candidate mutation	chr7:41 245 057	A	G	G/A	G/A	A/A	G/G	G/G	G/A	G/G	G/G
*Hepatoma Derived Growth Factor-Like 1*	*HDGFL1* predictive marker	Array marker	chr7:41 248 384	T	G	G/T	G/T	G/G	T/T	T/T	G/T	T/T	T/T

These dogs were sequenced at this gene location but had indels close to the variant site causing poor signal strength.

**Table 2:** Name and position of ten proposed risk variants, with representative genotypic array location, allele frequencies and reference and alternative alleles.

Gene	Variant	Position	Reference Allele Frequency CKCS ^a^	Reference Allele	Alternative Allele	Representative Genotyping Array Location	CKCS Observed Mean MAF (N = 180 Array)
*Nebulette*	*NEBL1*	chr2:11 816 535	0.00	A	G	chr2:11 822 980	0.02
*Nebulette*	*NEBL2*	chr2:11 823 576	0.00	C	T	chr2:11 832 538	0.02
*Nebulette*	*NEBL3*	chr2:11 979 724	0.00	G	A	chr2:11 979 724	0.02
*Nebulette*	*NEBL4*	chr2:12 082 890	0.99	T	C	chr2:12 085 928	0.05
*Nebulette*	*NEBL5*	chr2:12 165 498	0.94	A	T	chr2:12 174 951	0.05
*Nebulette*	*NEBL6*	chr2:12 567 546	0.03	A	G	chr2:12 567 760	0.01
*Latrophilin 2*	*LPHN2*	chr6:65 609 405	0.17	T	C	chr6:65 607 149	0.14
*Hepatoma Derived Growth Factor-Like 1*	*HDGFL1*	chr7:41 245 057	0.00	A	G	chr7:41 248 384	0.03
*Sorbin and SH3 Domain Containing 2*	*SORBS2*	chr16:45 026 823	0.25	C	T	chr16: 45 035 960	0.13
*Hydroxytryptamine Receptor 1F*	*HTR1F*	chr31:273 549	0.15	T	C	chr31:273 549	0.27

^a^ N = 35, Axelsson et al. Table 18 in supp info [6].

**Table 3:** Minor allele frequencies for proposed MMVD risk genes across 180 CKCS as ascertained by genotyping array markers.

Gene	Number of Markers	Mean Regional Minor Allele Frequency (±SD)	Accepted Status for Analysis
*NEBL*	91	0.01 (±0.01)	Potential fixation
*LPHN2*	97	0.19 (±0.18)	Polymorphic
*HDGFL1*	153	0.03 (±0.05)	Potential fixation
*SORBS2*	126	0.14 (±0.18)	Polymorphic
*HTR1F*	65	0.12 (±0.10)	Polymorphic

**Table 4:** Primers used for PCR reactions in preparation for sequencing at AGRF.

Variant	Forward Primer	Reverse Primer
*NEBL1*	GGAAGCAGGCTCAGACTCTC	AACCTGACCAGTCCTTGGTG
*NEBL2*	GCAGAAGGGCAACACTCTCT	TCTCTTTCTTTTGCCGCCCT
*NEBL3*	AGCCCTCCTTCTGTGCTTTA	CTCCAAGGAGCCATCACATT
*HDGFL1*	AGCACAGTCTCCCATCTCTC	CTCTCCAGGGGCTCTCTG

**Table 5:** Summary of phenotypic and echocardiographic data from 178 CKCS, including sex, ACVIM stage, age, LA:Ao and LVIDdN.

Variable	Total Cohort N = 178	NEBL1-3 Homozygotes N = 172	NEBL1-3 Heterozygotes N = 6
Total Males	80 (45%)	78 (45%)	2 (33%)
Total Females	98 (55%)	94 (55%)	4 (67%)
Total ACVIM Stage A	3 (2%)	2 (1%)	1 (17%)
Total ACVIM Stage B1	95 (53%)	91 (53%)	4 (67%)
Total ACVIM Stage B2	40 (22%)	39 (23%)	1 (17%)
Total ACVIM Stage C	36 (20%)	36 (21%)	0 (0%)
Total ACVIM Stage D	4 (2%)	4 (2%)	0 (0%)
Mean Age (±SD)	10 ± 1.96	10 ± 1.97	10 ± 1.87
Mean Age-Adjusted LA:Ao (±SD)	1.73 ± 0.62	1.74 ± 0.63	1.37 ± 0.21
Mean Age-Adjusted LVIDdN (±SD)	1.87 ± 0.46	1.88 ± 0.46	1.62 ± 0.16

**Table 6:** Reference allele frequencies for proposed MMVD risk variants across dog breeds and canids.

Gene Variant	Location (CF3)	REF ^a^	ALT ^b^	Reference Allele Frequency (Wolves, N = 8) ^c^	Reference Allele Frequency (Domestic dogs, N = 582) ^c^	Reference Allele Frequency (CKCS, N = 4) ^c^	Reference Allele Frequency (CKCS, N = 96) ^d^
*NEBL1*	chr2:11816535	A	G	0.81	0.56	0.13	
*NEBL2*	chr2:11823576	C	T	0.69	0.59	0.00	
*NEBL3*	chr2:11979724	G	A	1.00	0.85	0.13	0.01
*NEBL4*	chr2:12082890	T	C	0.06	0.28	1.00	
*NEBL5*	chr2:12165498	A	T	0.06	0.21	1.00	
*NEBL6*	chr2:12567546	A	G	0.75	0.84	0.00	
*LPHN2*	chr6:65609405	T	C	1.00	0.95	0.38	
*HDGFL1*	chr7:41245057	A	G	1.00	0.72	0.00	0.01
*SORBS2*	chr16:45026823	C	T	1.00	0.99	0.50	
*HTR1F*	chr31:273549	T	C	1.00	0.96	0.50	

^a^ REF: Reference allele canfam3.1 Boxer assembly; ^b^ ALT: Alternate allele canfam3.1 Boxer assembly; ^c^ Jagannathan et al., 2019 [17]; ^d^ Ancot et al., 2018 [18].

**Table 7:** Mean (±SD) values for LA:Ao and LVIDdN for heterozygous vs. homozygous dogs at *HDGFL1* and *NEBL1-6* variant array markers ^1^.

Variant (Representative Array Marker)	Mean Homozygous Cohort	Mean Heterozygous Cohort	F-Test F Value	F-Test Critical F value	T-Test T Statistic
Age-Adjusted LA:Ao					
*NEBL1,2,3*	1.74 ± 0.63	1.37 ± 0.21	9.38 **	4.39 **	3.82 **
*NEBL4*	1.76 ± 0.64	1.41 ± 0.26 ^1^	6.09 **	2.05 **	4.41 **
*NEBL5*	1.76 ± 0.64	1.41 ± 0.26 ^1^	6.09 **	2.05 **	4.41 **
*NEBL6*	1.73 ± 0.63	1.53 ± 0.16 ^1^	16.11	19.49	1.99
*HDGFL1*	1.72 ± 0.62	1.96 ± 0.70 ^1^	1.26	1.99	1.02
Age-Adjusted LVIDdN					
*NEBL1,2,3*	1.88 ± 0.46	1.62 ± 0.16 ^1^	8.64 **	4.39 *	3.52 **
*NEBL4*	1.89 ± 0.47	1.69 ± 0.23 ^1^	4.06 **	2.05 **	3.02 **
*NEBL5*	1.89 ± 0.47	1.69 ± 0.23 ^1^	4.06 **	2.05 **	3.02 **
*NEBL6*	1.87 ± 0.46	1.66 ± 0.26 ^1^	3.22	19.49	1.41
*HDGFL1*	1.87 ± 0.45	1.92 ± 0.55 ^1^	1.49	1.99	0.26

^1^*p*-value significance markers ** < 0.01, * ≤ 0.01.

## Data Availability

Not applicable.

## Supplementary Materials

The following supporting information can be downloaded at: https://www.mdpi.com/article/10.3390/genes13122292/s1, Table S1: Phenotypic characteristics of 178 Australian CKCS dogs, including echocardiographic heart measurements, Table S2: PLINK -freq data for for MMVD candidate variant gene regions SNPs from 180 Australian CKCS, Table S3: PLINK -freqx data for MMVD candidate variant gene regions SNPs from 180 Australian CKCS.
