# Supplementary material for: Genetic Variants at the Nebulette Locus Are Associated with Myxomatous Mitral Valve Disease Severity in Cavalier King Charles Spaniels
Source: Genes (Basel). 2022 Dec 5;13(12):2292. doi: 10.3390/genes13122292 (PMC9778376; doi:10.3390/genes13122292)
Supplement: Supplementary file 1 [file genes-13-02292-s001.zip › genes-2055830-Supplementary.pdf]

Supplementary Information

**Table S1.** Phenotypic characteristics of 178 Australian CKCS dogs, including echocardiographic heart measurements.

| (USCF) | Sex | Age (years) | Bodyweight (kg) | ACVIM | LA/Ao | LVIDdn | LA/Ao age adjusted | LVIDdn age adjusted |
|---|---|---|---|---|---|---|---|---|
| 2970 | M | 8 | 12 | B2 | 1.66 | 1.75 | 1.69 | 1.76 |
| 2971 | F | 10 | 9 | B1 | 1.59 | 1.58 | 1.55 | 1.51 |
| 2972 | M | 9 | 9 | B2 | 2.00 | 2.41 | 1.98 | 2.38 |
| 2973 | F | 10 | 6.3 | C | 1.85 | 2.08 | 1.80 | 2.01 |
| 2974 | M | 14 | 8 | B2 | 1.89 | 2.25 | 1.74 | 2.08 |
| 2975 | M | 14 | 8.3 | B1 | 1.13 | 1.66 | 0.99 | 1.50 |
| 2976 | M | 9 | 9.5 | C | 2.11 | 2.38 | 2.09 | 2.34 |
| 2977 | F | 9 | 7 | D | 3.00 | 2.92 | 2.99 | 2.89 |
| 2978 | F | 8 | 8.2 | B1 | 1.44 | 2.15 | 1.45 | 2.14 |
| 2979 | F | 9 | 9.4 | C | 3.63 | 3.07 | 3.62 | 3.04 |
| 2980 | F | 11 | 10 | C | 2.29 | 2.29 | 2.23 | 2.21 |
| 2981 | F | 10 | 8 | B1 | 1.57 | 1.84 | 1.52 | 1.77 |
| 2982 | F | 10 | 8 | B1 | 1.33 | 1.84 | 1.30 | 1.79 |
| 2983 | M | 14 | 7 | B1 | 1.89 | 1.66 | 1.74 | 1.49 |
| 2984 | F | 13 | 6 | B1 | 1.24 | 1.73 | 1.10 | 1.57 |
| 2985 | F | 12 | 8 | C | 2.31 | 2.61 | 2.20 | 2.48 |
| 2986 | F | 11 | 7.5 | B1 | 1.25 | 1.65 | 1.18 | 1.56 |
| 2987 | M | 8 | 10.8 | D | 3.04 | 2.41 | 3.06 | 2.41 |
| 2988 | F | 12 | 11.3 | B1 | 1.79 | 1.57 | 1.70 | 1.46 |
| 2989 | M | 8 | 8.8 | B1 | 1.51 | 1.55 | 1.53 | 1.55 |
| 2990 | M | 9 | 11.8 | B1 | 1.87 | 1.63 | 1.87 | 1.61 |
| 2991 | M | 13 | 9 | B1 | 1.17 | 1.64 | 1.04 | 1.49 |
| 2992 | F | 10 | 8.4 | C | 2.22 | 2.09 | 2.19 | 2.04 |
| 2993 | F | 10 | 8.5 | B1 | 1.41 | 1.51 | 1.37 | 1.45 |
| 2994 | M | 16 | 8.8 | B1 | 1.09 | 1.46 | 0.86 | 1.21 |
| 2995 | M | 10 | 7.8 | C | 2.38 | 2.43 | 2.34 | 2.37 |
| 2996 | F | 6 | 10.9 | C | 2.90 | 2.48 | 2.97 | 2.53 |
| 2997 | M | 8 | 11.2 | B2 | 2.39 | 2.31 | 2.41 | 2.31 |
| 2998 | M | 10 | 9.3 | D | 3.56 | 2.92 | 3.51 | 2.85 |
| 3246 | F | 12 | 7.7 | B1 | 1.43 | 1.88 | 1.34 | 1.77 |
| 3247 | M | 12 | 8.97 | C | 2.30 | 2.32 | 2.19 | 2.19 |
| 3248 | F | 10 | 9.2 | B2 | 1.61 | 1.83 | 1.58 | 1.78 |
| 3249 | F | 8 | 6.9 | B2 | 1.98 | 2.08 | 2.01 | 2.09 |
| 3250 | F | 8 | 4.8 | B1 | 1.08 | 1.35 | 1.11 | 1.36 |
| 3251 | F | 9 | 7.1 | B1 | 1.49 | 1.80 | 1.47 | 1.76 |
| 3252 | M | 9 | 7.8 | B1 | 1.23 | 1.48 | 1.23 | 1.46 |
| 3253 | F | 10 | 8.5 | B1 | 1.21 | 1.60 | 1.16 | 1.53 |
| 3254 | F | 7 | 9.1 | B1 | 1.43 | 1.20 | 1.47 | 1.22 |
| 3255 | F | 15 | 7.4 | B2 | 1.80 | 1.94 | 1.62 | 1.74 |

| | | | | | | | | |
|---|---|---|---|---|---|---|---|---|
| 3256 | F | 9 | 7.35 | B2 | 1.67 | 1.87 | 1.65 | 1.83 |
| 3257 | M | 9 | 8.35 | B1 | 1.37 | 1.57 | 1.35 | 1.53 |
| 3258 | M | 9 | 8.6 | C | 2.31 | 2.32 | 2.31 | 2.30 |
| 3259 | M | 8 | 8.93 | B1 | 1.29 | 1.31 | 1.31 | 1.31 |
| 3260 | F | 8 | 7.8 | B1 | 1.21 | 1.68 | 1.23 | 1.68 |
| 3261 | M | 11 | 8.52 | B1 | 1.67 | 1.51 | 1.61 | 1.43 |
| 3262 | M | 11 | 7.56 | B2 | 2.16 | 2.26 | 2.10 | 2.18 |
| 3265 | F | 7 | 7.9 | C | 2.29 | 2.56 | 2.33 | 2.58 |
| 3266 | M | 7 | 10.6 | B1 | 1.21 | 1.76 | 1.25 | 1.78 |
| 3267 | M | 15 | 9.5 | B1 | 1.53 | 2.04 | 1.33 | 1.82 |
| 3268 | M | 12 | 9.06 | B1 | 1.53 | 2.06 | 1.42 | 1.93 |
| 3269 | M | 10 | 9.3 | B1 | 1.41 | 1.87 | 1.36 | 1.80 |
| 3270 | F | 13 | 11 | B2 | 2.54 | 2.19 | 2.42 | 2.05 |
| 3271 | F | 12 | 8 | B2 | 1.93 | 2.33 | 1.84 | 2.22 |
| 3272 | M | 9 | 8.2 | B2 | 2.16 | 2.42 | 2.14 | 2.38 |
| 3273 | M | 11 | 9.37 | B2 | 2.06 | 2.23 | 2.00 | 2.15 |
| 3274 | M | 9 | 9.23 | B1 | 1.55 | 1.91 | 1.53 | 1.87 |
| 3275 | M | 12 | 8.5 | B1 | 1.52 | 1.87 | 1.43 | 1.76 |
| 3276 | M | 8 | 11.6 | B2 | 1.80 | 2.03 | 1.81 | 2.02 |
| 3277 | F | 13 | 8.2 | B1 | 1.20 | 1.67 | 1.09 | 1.54 |
| 3278 | M | 11 | 11.5 | C | 2.41 | 2.52 | 2.33 | 2.42 |
| 3280 | F | 9 | 7.4 | C | 2.40 | 2.35 | 2.38 | 2.31 |
| 3281 | M | 11 | 8 | C | 2.61 | 2.30 | 2.54 | 2.21 |
| 3282 | M | 10 | 9 | B1 | 1.47 | 2.13 | 1.42 | 2.06 |
| 3283 | F | 10 | 7.7 | B2 | 1.73 | 1.70 | 1.68 | 1.63 |
| 3284 | F | 12 | 7.9 | B1 | 1.24 | 1.63 | 1.14 | 1.51 |
| 3285 | F | 10 | 8.8 | B1 | 1.40 | 1.92 | 1.35 | 1.85 |
| 3286 | M | 9 | 8.1 | C | 3.59 | 2.81 | 3.59 | 2.79 |
| 3287 | M | 8 | 15.2 | C | 1.98 | 2.02 | 1.99 | 2.01 |
| 3288 | M | 9 | 9.9 | D | 3.58 | 2.75 | 3.57 | 2.72 |
| 3289 | M | 11 | 11.1 | C | 2.95 | 2.11 | 2.88 | 2.02 |
| 3290 | F | 13 | 9 | B1 | 1.43 | 1.03 | 1.29 | 0.87 |
| 3291 | F | 9 | 8.7 | C | 3.47 | 2.21 | 3.47 | 2.19 |
| 3292 | F | 8 | 11.6 | C | 2.51 | 2.21 | 2.52 | 2.20 |
| 3293 | F | 11 | 9.8 | C | 2.12 | 2.47 | 2.06 | 2.39 |
| 3294 | F | 11 | 8.5 | B2 | 2.08 | 1.76 | 2.00 | 1.66 |
| 3295 | M | 8 | 11.6 | A | 1.13 | 1.56 | 1.15 | 1.56 |
| 3296 | F | 9 | 10 | C | 3.00 | 2.77 | 2.73 | 2.73 |
| 3297 | M | 10 | 14 | B1 | 1.11 | 1.06 | 1.07 | 1.00 |
| 3298 | M | 10 | 8 | B2 | 2.17 | 2.28 | 2.14 | 2.23 |
| 3299 | F | 11 | 8.3 | B1 | 1.00 | 1.78 | 0.92 | 1.68 |
| 3300 | F | 9 | 10.2 | B2 | 1.94 | 1.88 | 1.92 | 1.84 |
| 3301 | M | 9 | 7.7 | B2 | 2.87 | 2.64 | 2.85 | 2.60 |
| 3302 | M | 8 | 9.2 | B1 | 1.23 | 1.62 | 1.24 | 1.61 |
| 3303 | F | 13 | 7.1 | B1 | 1.39 | 1.11 | 1.27 | 0.97 |

| | | | | | | | | |
|---|---|---|---|---|---|---|---|---|
| 3304 | M | 10 | 6.2 | B1 | 1.25 | 1.54 | 1.21 | 1.48 |
| 3305 | M | 11 | 8.7 | C | 2.80 | 2.74 | 2.73 | 2.65 |
| 3306 | F | 12 | 10 | B1 | 1.06 | 1.63 | 0.96 | 1.51 |
| 3308 | M | 9 | 9.2 | B2 | 1.76 | 1.73 | 1.75 | 1.70 |
| 3309 | F | 9 | 5 | B1 | 1.39 | 1.87 | 1.38 | 1.84 |
| 3310 | F | 8 | 8 | B1 | 1.41 | 1.73 | 1.42 | 1.72 |
| 3311 | F | 14 | 8 | B2 | 1.63 | 2.17 | 1.49 | 2.01 |
| 3312 | F | 8 | 9.3 | B1 | 1.40 | 1.32 | 1.43 | 1.33 |
| 3313 | M | 14 | 9.8 | B1 | 1.35 | 1.60 | 1.19 | 1.42 |
| 3314 | M | 8 | 8.9 | C | 2.50 | 2.60 | 2.53 | 2.61 |
| 3315 | M | 11 | 10.1 | C | 1.80 | 1.92 | 1.82 | 1.80 |
| 3316 | F | 12 | 7.6 | B2 | 1.89 | 2.26 | 1.81 | 2.16 |
| 3317 | F | 8 | 8 | B1 | 1.42 | 1.48 | 1.44 | 1.48 |
| 3319 | M | 12 | 9.5 | B2 | 1.74 | 1.85 | 1.65 | 1.74 |
| 3320 | F | 14 | 7 | B2 | 2.44 | 2.39 | 2.27 | 2.20 |
| 3321 | F | 12 | 9 | B2 | 1.84 | 2.09 | 1.74 | 1.97 |
| 3322 | M | 10 | 7.5 | B1 | 1.41 | 1.57 | 1.36 | 1.50 |
| 3323 | M | 10 | 8 | B1 | 1.27 | 1.67 | 1.24 | 1.62 |
| 3324 | F | 9 | 7.2 | B1 | 1.13 | 1.43 | 1.12 | 1.40 |
| 3325 | F | 9 | 8.5 | B1 | 1.52 | 2.15 | 1.51 | 2.12 |
| 3326 | M | 10 | 9 | B1 | 1.44 | 1.74 | 1.41 | 1.69 |
| 3327 | M | 7 | 9 | B1 | 1.48 | 1.72 | 1.52 | 1.74 |
| 3328 | M | 11 | 11 | B2 | 1.78 | 2.35 | 1.70 | 2.25 |
| 3329 | M | 13 | 8.5 | B2 | 1.94 | 2.00 | 1.81 | 1.85 |
| 3330 | F | 10 | 7 | B2 | 1.78 | 2.08 | 1.73 | 2.01 |
| 3331 | F | 11 | 9 | B1 | 1.55 | 1.89 | 1.49 | 1.81 |
| 3332 | F | 10 | 7 | B1 | 1.31 | 1.74 | 1.26 | 1.67 |
| 3333 | F | 8 | 9 | B1 | 1.21 | 1.57 | 1.24 | 1.58 |
| 3334 | F | 10 | 9.5 | B1 | 1.51 | 1.78 | 1.48 | 1.73 |
| 3335 | F | 11 | 8 | B1 | 1.50 | 1.86 | 1.42 | 1.76 |
| 3336 | F | 10 | 8.3 | C | 2.34 | 2.37 | 2.30 | 2.31 |
| 3337 | M | 7 | 7 | C | 2.97 | 4.12 | 3.01 | 4.14 |
| 3338 | M | 9 | 14 | B2 | 1.77 | 1.74 | 1.77 | 1.72 |
| 3341 | F | 10 | 7.1 | C | 3.11 | 2.79 | 3.07 | 2.73 |
| 3371 | M | 8 | 9.2 | C | 3.48 | 2.67 | 3.50 | 2.67 |
| 3372 | M | 12 | 14.0 | B1 | 1.01 | 1.05 | 0.94 | 0.90 |
| 3373 | M | 11 | 16.3 | B1 | 1.49 | 1.86 | 1.41 | 1.76 |
| 3405 | M | 12 | 8 | B2 | 1.83 | 2.17 | 1.72 | 2.04 |
| 3406 | M | 16 | 11.7 | B1 | 1.31 | 1.39 | 1.11 | 1.17 |
| 3407 | F | 11 | 6.13 | B1 | 1.26 | 1.55 | 1.20 | 1.47 |
| 3408 | F | 14 | 7.5 | B1 | 1.49 | 1.97 | 1.35 | 1.81 |
| 3409 | F | 13 | 9 | B1 | 1.60 | 1.68 | 1.47 | 1.53 |
| 3410 | F | 9 | 7.9 | B1 | 1.30 | 1.74 | 1.29 | 1.71 |
| 3411 | F | 15 | 9 | B1 | 1.40 | 1.54 | 1.21 | 1.33 |
| 3412 | F | 12 | 7.7 | B1 | 1.38 | 1.40 | 1.29 | 1.29 |

| | | | | | | | | |
|---|---|---|---|---|---|---|---|---|
| 3414 | F | 11 | 7.5 | B2 | 1.86 | 2.16 | 1.78 | 2.06 |
| 3416 | F | 9 | 17.3 | B1 | 1.04 | 1.30 | 1.02 | 1.26 |
| 3417 | F | 10 | 8.5 | B1 | 1.40 | 1.55 | 1.36 | 1.49 |
| 3418 | M | 13 | 8 | B1 | 1.32 | 1.69 | 1.20 | 1.55 |
| 3419 | F | 11 | 10 | B1 | 1.24 | 1.72 | 1.19 | 1.65 |
| 3420 | F | 10 | 11.5 | B1 | 1.28 | 1.42 | 1.24 | 1.36 |
| 80259 | F | 10 | 10 | B1 | 1.48 | 1.75 | 1.44 | 1.69 |
| 80260 | M | 11 | 9 | B1 | 1.04 | 2.05 | 0.98 | 1.97 |
| 80261 | F | 11 | 9 | B2 | 1.95 | 2.43 | 1.90 | 2.36 |
| 80262 | M | 9 | 8.4 | B2 | 1.98 | 2.27 | 1.97 | 2.24 |
| 80263 | F | 10 | 8.5 | B1 | 1.71 | 1.68 | 1.66 | 1.61 |
| 80265 | M | 9 | 12.8 | C | 2.59 | 2.52 | 2.58 | 2.49 |
| 80266 | M | 14 | 8.0 | C | 2.43 | 1.90 | 2.28 | 1.73 |
| 80267 | F | 14 | 6.5 | B1 | 1.54 | 1.67 | 1.39 | 1.50 |
| 80268 | F | 9 | 6.7 | B2 | 2.20 | 2.46 | 2.20 | 2.44 |
| 80269 | F | 10 | 7.8 | B2 | 1.71 | 1.87 | 1.68 | 1.82 |
| 80270 | F | 8 | 9.0 | B2 | 1.61 | 1.79 | 1.62 | 1.78 |
| 80271 | M | 13 | 8.0 | B1 | 1.19 | 1.77 | 1.06 | 1.62 |
| 80272 | F | 11 | 7.5 | B1 | 1.11 | 1.41 | 1.03 | 1.31 |
| 80273 | F | 9 | 7.0 | B1 | 1.24 | 2.24 | 1.24 | 2.22 |
| 80274 | M | 8 | 10.6 | C | 2.60 | 2.28 | 2.64 | 2.30 |
| 80275 | F | 9 | 11.0 | B1 | 1.40 | 1.69 | 1.40 | 1.67 |
| 80276 | F | 10 | 7.0 | B1 | 1.38 | 1.22 | 1.34 | 1.16 |
| 80277 | M | 8 | 7.8 | C | 3.70 | 2.90 | 3.72 | 2.90 |
| 80278 | M | 9 | 10.6 | C | 2.94 | 2.15 | 2.93 | 2.12 |
| 80279 | M | 11 | 10.8 | B1 | 1.36 | 1.76 | 1.28 | 1.66 |
| 80280 | F | 11 | 9.0 | B1 | 1.42 | 1.78 | 1.35 | 1.69 |
| 80281 | M | 8 | 8.0 | B1 | 1.35 | 1.74 | 1.36 | 1.73 |
| 80282 | M | 9 | 8.0 | B2 | 1.94 | 2.42 | 1.92 | 2.38 |
| 80283 | F | 9 | 10.0 | B1 | 1.15 | 1.81 | 1.16 | 1.80 |
| 80284 | F | 8 | 7.5 | B1 | 1.36 | 1.86 | 1.37 | 1.85 |
| 80285 | F | 12 | 10.0 | B1 | 1.09 | 1.65 | 1.00 | 1.54 |
| 80286 | F | 10 | 6.7 | C | 1.76 | 2.09 | 1.73 | 2.04 |
| 80287 | M | 8 | 10.0 | B1 | 1.44 | 1.62 | 1.45 | 1.61 |
| 80288 | F | 8 | 10.0 | B1 | 1.21 | 1.69 | 1.22 | 1.68 |
| 80289 | F | 11 | 7.5 | B1 | 1.16 | 1.41 | 1.09 | 1.32 |
| 80290 | F | 9 | 7.1 | B1 | 1.43 | 1.58 | 1.41 | 1.54 |
| 80291 | F | 14 | 9.6 | B1 | 1.34 | 1.50 | 1.17 | 1.31 |
| 80292 | M | 8 | 8.5 | B1 | 1.47 | 1.93 | 1.49 | 1.93 |
| 80293 | F | 10 | 8.0 | B2 | 1.65 | 2.01 | 1.62 | 1.96 |
| 80294 | F | 9 | 7.5 | A | 1.35 | 1.38 | 1.35 | 1.36 |
| 80295 | M | 9 | 8.5 | B2 | 1.87 | 1.79 | 1.85 | 1.75 |
| 80296 | F | 9 | 10.4 | B1 | 1.59 | 2.19 | 1.58 | 2.17 |
| 80297 | F | 15 | 6 | C | 1.99 | 2.38 | 1.79 | 2.16 |
| 80298 | F | 9 | 8 | B1 | 1.54 | 1.73 | 1.52 | 1.69 |

| | | | | | | | | |
|---|---|---|---|---|---|---|---|---|
| 80299 | M | 10 | 11 | B1 | 1.49 | 1.59 | 1.45 | 1.52 |
| 80300 | M | 9 | 12 | B2 | 1.74 | 1.99 | 1.73 | 1.97 |
| 80301 | F | 9 | 10 | A | 1.30 | 1.36 | 1.29 | 1.33 |
| 80302 | M | 10 | 13.7 | C | 1.82 | 2.15 | 1.79 | 2.10 |

**Table S2.** PLINK -freq data for for MMVD candidate variant gene regions SNPs from 180 Australian CKCS.

| CHR | SNP | A1 | A2 | MAF | NCHROBS |
|---|---|---|---|---|---|
| 2 | 2.11654206 | 0 | T | 0 | 360 |
| 2 | 2.11662236 | 0 | A | 0 | 360 |
| 2 | 2.1167411 | 0 | A | 0 | 360 |
| 2 | 2.11689791 | 0 | T | 0 | 360 |
| 2 | 2.11695641 | 0 | A | 0 | 360 |
| 2 | 2.11707593 | 0 | T | 0 | 360 |
| 2 | 2.11717132 | T | C | 0.01944 | 360 |
| 2 | 2.11730035 | C | T | 0.01944 | 360 |
| 2 | 2.11739358 | 0 | T | 0 | 360 |
| 2 | 2.11751842 | 0 | C | 0 | 360 |
| 2 | 2.11764533 | A | G | 0.01944 | 360 |
| 2 | 2.11774349 | A | G | 0.01944 | 360 |
| 2 | 2.11787735 | G | C | 0.03611 | 360 |
| 2 | 2.11793471 | G | A | 0.03611 | 360 |
| 2 | 2.11799338 | G | A | 0.03611 | 360 |
| 2 | 2.118124 | 0 | G | 0 | 360 |
| 2 | 2.1182298 | A | G | 0.01667 | 360 |
| 2 | 2.11832538 | G | A | 0.01667 | 360 |
| 2 | 2.11843668 | 0 | A | 0 | 360 |
| 2 | 2.11855019 | 0 | C | 0 | 360 |
| 2 | 2.11871891 | A | G | 0.01744 | 344 |
| 2 | 2.11878409 | A | G | 0.03611 | 360 |
| 2 | 2.11900062 | 0 | G | 0 | 360 |
| 2 | 2.11901965 | 0 | T | 0 | 360 |
| 2 | 2.11914478 | 0 | C | 0 | 360 |
| 2 | 2.11924282 | A | C | 0.03611 | 360 |
| 2 | 2.11937039 | 0 | A | 0 | 360 |
| 2 | 2.11946899 | 0 | G | 0 | 360 |
| 2 | 2.11962479 | T | G | 0.01667 | 360 |
| 2 | 2.11979724 | G | A | 0.01667 | 360 |
| 2 | 2.11989819 | T | C | 0.01667 | 360 |
| 2 | 2.12002367 | C | A | 0.01667 | 360 |
| 2 | 2.12012688 | T | C | 0.05 | 360 |
| 2 | 2.12019966 | C | T | 0.03056 | 360 |
| 2 | 2.12030635 | C | T | 0.03056 | 360 |
| 2 | 2.12039245 | 0 | A | 0 | 360 |
| 2 | 2.12053055 | 0 | T | 0 | 360 |

| | | | | | |
|---|---|---|---|---|---|
| 2 | 2.12063981 | C | T | 0.04722 | 360 |
| 2 | 2.12078166 | 0 | C | 0 | 360 |
| 2 | 2.12085928 | A | G | 0.04722 | 360 |
| 2 | 2.12102288 | 0 | C | 0 | 360 |
| 2 | 2.12108037 | C | T | 0.04722 | 360 |
| 2 | 2.12122303 | A | G | 0.04722 | 360 |
| 2 | 2.12126839 | T | A | 0.01667 | 360 |
| 2 | 2.12131129 | 0 | C | 0 | 360 |
| 2 | 2.12141216 | 0 | G | 0 | 360 |
| 2 | 2.1214462 | 0 | A | 0 | 360 |
| 2 | 2.12153524 | A | G | 0.01667 | 360 |
| 2 | 2.12162327 | 0 | C | 0 | 352 |
| 2 | 2.12174951 | C | A | 0.04722 | 360 |
| 2 | 2.12184406 | A | G | 0.03056 | 360 |
| 2 | 2.12192987 | G | A | 0.04722 | 360 |
| 2 | 2.1220313 | T | G | 0.01944 | 360 |
| 2 | 2.1221131 | C | A | 0.01944 | 360 |
| 2 | 2.12230658 | T | C | 0.01944 | 360 |
| 2 | 2.12234249 | A | T | 0.002778 | 360 |
| 2 | 2.12245988 | C | T | 0.002778 | 360 |
| 2 | 2.1225981 | T | C | 0.01667 | 360 |
| 2 | 2.12269676 | C | A | 0.002778 | 360 |
| 2 | 2.12283473 | A | G | 0.01944 | 360 |
| 2 | 2.12306926 | A | G | 0.01944 | 360 |
| 2 | 2.12318063 | G | A | 0.01944 | 360 |
| 2 | 2.12326789 | T | C | 0.01944 | 360 |
| 2 | 2.12347206 | G | A | 0.01944 | 360 |
| 2 | 2.12349813 | T | C | 0.01667 | 360 |
| 2 | 2.12361062 | C | A | 0.01667 | 360 |
| 2 | 2.12376723 | G | T | 0.002778 | 360 |
| 2 | 2.12386738 | A | G | 0.002778 | 360 |
| 2 | 2.12396395 | T | A | 0.002778 | 360 |
| 2 | 2.12413114 | T | G | 0.002778 | 360 |
| 2 | 2.12418462 | T | G | 0.002778 | 360 |
| 2 | 2.12434761 | 0 | A | 0 | 360 |
| 2 | 2.12442689 | 0 | G | 0 | 360 |
| 2 | 2.12453483 | T | C | 0.002809 | 356 |
| 2 | 2.12469577 | C | T | 0.002778 | 360 |
| 2 | 2.12478569 | A | G | 0.002778 | 360 |
| 2 | 2.12495431 | C | T | 0.002778 | 360 |
| 2 | 2.1250943 | 0 | A | 0 | 360 |
| 2 | 2.12512843 | T | C | 0.005587 | 358 |
| 2 | 2.12526445 | C | T | 0.002809 | 356 |
| 2 | 2.12533482 | C | T | 0.008333 | 360 |
| 2 | 2.12544856 | T | C | 0.008333 | 360 |

| | | | | | |
|---|---|---|---|---|---|
| 2 | 2.12565297 | C | T | 0.008333 | 360 |
| 2 | 2.1256776 | C | T | 0.008333 | 360 |
| 2 | 2.12585933 | T | C | 0.002778 | 360 |
| 2 | 2.12597489 | T | C | 0.008333 | 360 |
| 2 | 2.12611546 | G | A | 0.008333 | 360 |
| 2 | 2.12613634 | C | T | 0.008333 | 360 |
| 2 | 2.12624913 | 0 | A | 0 | 360 |
| 2 | 2.1264288 | 0 | T | 0 | 360 |
| 2 | 2.12643962 | C | G | 0.008333 | 360 |
| 6 | 6.6510114 | A | G | 0.1639 | 360 |
| 6 | 6.65112557 | T | C | 0.1056 | 360 |
| 6 | 6.65126636 | G | A | 0.075 | 360 |
| 6 | 6.65136211 | G | A | 0.07542 | 358 |
| 6 | 6.6515326 | 0 | C | 0 | 360 |
| 6 | 6.65160298 | 0 | C | 0 | 360 |
| 6 | 6.65174782 | A | G | 0.01389 | 360 |
| 6 | 6.65180856 | T | C | 0.08333 | 360 |
| 6 | 6.65195796 | 0 | A | 0 | 360 |
| 6 | 6.65209291 | G | A | 0.005556 | 360 |
| 6 | 6.65221796 | G | A | 0.1 | 360 |
| 6 | 6.6523303 | C | A | 0.2028 | 360 |
| 6 | 6.65246451 | C | T | 0.1778 | 360 |
| 6 | 6.65260015 | T | C | 0.4806 | 360 |
| 6 | 6.65270131 | T | C | 0.4444 | 360 |
| 6 | 6.65270332 | A | G | 0.4444 | 360 |
| 6 | 6.65276297 | T | A | 0.4528 | 360 |
| 6 | 6.65287462 | G | T | 0.4528 | 360 |
| 6 | 6.65298372 | A | G | 0.3583 | 360 |
| 6 | 6.65310259 | G | A | 0.09444 | 360 |
| 6 | 6.65319953 | G | A | 0.4278 | 360 |
| 6 | 6.65322941 | G | A | 0.01389 | 360 |
| 6 | 6.65332532 | T | C | 0.4583 | 360 |
| 6 | 6.65344346 | C | T | 0.4528 | 360 |
| 6 | 6.65348597 | A | G | 0.3583 | 360 |
| 6 | 6.65362922 | A | G | 0.3583 | 360 |
| 6 | 6.65386534 | A | G | 0.4417 | 360 |
| 6 | 6.65389473 | A | G | 0.3667 | 360 |
| 6 | 6.65406362 | C | T | 0.008333 | 360 |
| 6 | 6.65418337 | T | G | 0.3167 | 360 |
| 6 | 6.65423779 | A | G | 0.008523 | 352 |
| 6 | 6.65435312 | C | T | 0.3222 | 360 |
| 6 | 6.65447904 | T | C | 0.3139 | 360 |
| 6 | 6.65449088 | C | T | 0.3194 | 360 |
| 6 | 6.65463236 | T | G | 0.3278 | 360 |
| 6 | 6.65470988 | T | C | 0.008333 | 360 |

| | | | | | |
|---|---|---|---|---|---|
| 6 | 6.65480518 | 0 | G | 0 | 360 |
| 6 | 6.65496597 | T | C | 0.008333 | 360 |
| 6 | 6.65508132 | 0 | A | 0 | 360 |
| 6 | 6.65523436 | G | A | 0.175 | 360 |
| 6 | 6.65525864 | A | C | 0.1 | 360 |
| 6 | 6.65540825 | 0 | A | 0 | 360 |
| 6 | 6.65549874 | C | T | 0.1889 | 360 |
| 6 | 6.65567826 | 0 | C | 0 | 360 |
| 6 | 6.65578336 | A | G | 0.03313 | 332 |
| 6 | 6.65587233 | C | T | 0.1889 | 360 |
| 6 | 6.65595431 | 0 | G | 0 | 360 |
| 6 | 6.65596861 | C | T | 0.225 | 360 |
| 6 | 6.65598045 | 0 | C | 0 | 358 |
| 6 | 6.65607149 | A | G | 0.1361 | 360 |
| 6 | 6.65618215 | T | C | 0.08889 | 360 |
| 6 | 6.65633801 | A | C | 0.3 | 360 |
| 6 | 6.65645598 | 0 | A | 0 | 354 |
| 6 | 6.65656909 | T | C | 0.1139 | 360 |
| 6 | 6.65674786 | A | G | 0.005556 | 360 |
| 6 | 6.65675778 | C | A | 0.2949 | 356 |
| 6 | 6.65686888 | T | C | 0.1028 | 360 |
| 6 | 6.65705271 | C | A | 0.1083 | 360 |
| 6 | 6.65720395 | C | A | 0.1194 | 360 |
| 6 | 6.65723007 | G | A | 0.005556 | 360 |
| 6 | 6.65737294 | T | C | 0.2583 | 360 |
| 6 | 6.65746902 | G | A | 0.1083 | 360 |
| 6 | 6.65755813 | 0 | G | 0 | 360 |
| 6 | 6.65767474 | G | A | 0.2083 | 360 |
| 6 | 6.65781207 | A | G | 0.2778 | 360 |
| 6 | 6.65791139 | 0 | C | 0 | 360 |
| 6 | 6.65804588 | G | A | 0.4833 | 360 |
| 6 | 6.65822393 | A | G | 0.005988 | 334 |
| 6 | 6.65823535 | G | A | 0.4833 | 360 |
| 6 | 6.65823601 | G | A | 0.4833 | 360 |
| 6 | 6.65827291 | 0 | G | 0 | 360 |
| 6 | 6.65842149 | 0 | G | 0 | 360 |
| 6 | 6.65849064 | G | T | 0.3904 | 356 |
| 6 | 6.6586633 | G | A | 0.3904 | 356 |
| 6 | 6.65870908 | G | T | 0.4639 | 360 |
| 6 | 6.65882687 | A | G | 0.3444 | 360 |
| 6 | 6.65904868 | 0 | G | 0 | 360 |
| 6 | 6.6591498 | A | G | 0.3972 | 360 |
| 6 | 6.65917393 | T | C | 0.3972 | 360 |
| 6 | 6.65928843 | A | G | 0.3389 | 360 |
| 6 | 6.65928931 | C | T | 0.3389 | 360 |

| | | | | | |
|---|---|---|---|---|---|
| 6 | 6.65946566 | 0 | A | 0 | 360 |
| 6 | 6.65951982 | 0 | T | 0 | 360 |
| 6 | 6.65954545 | C | T | 0.06944 | 360 |
| 6 | 6.6596638 | 0 | C | 0 | 360 |
| 6 | 6.65975858 | A | C | 0.3944 | 360 |
| 6 | 6.65994724 | G | A | 0.4889 | 360 |
| 6 | 6.65998281 | G | A | 0.4167 | 360 |
| 6 | 6.66013403 | A | G | 0.4056 | 360 |
| 6 | 6.66024215 | C | T | 0.4556 | 360 |
| 6 | 6.66035679 | G | A | 0.06389 | 360 |
| 6 | 6.66048377 | T | C | 0.006061 | 330 |
| 6 | 6.66055784 | A | G | 0.005556 | 360 |
| 6 | 6.66070005 | 0 | C | 0 | 360 |
| 6 | 6.66088204 | 0 | G | 0 | 360 |
| 6 | 6.66098802 | 0 | G | 0 | 360 |
| 6 | 6.66106577 | C | T | 0.04444 | 360 |
| 7 | 7.40767427 | G | T | 0.07184 | 348 |
| 7 | 7.40778971 | C | T | 0.2 | 10 |
| 7 | 7.40801645 | G | A | 0.2 | 10 |
| 7 | 7.40812558 | T | C | 0.05966 | 352 |
| 7 | 7.40815795 | 0 | C | 0 | 360 |
| 7 | 7.40819079 | C | G | 0.08947 | 190 |
| 7 | 7.40823701 | T | A | 0.07222 | 360 |
| 7 | 7.40824538 | G | A | 0.002778 | 360 |
| 7 | 7.40827852 | 0 | A | 0 | 360 |
| 7 | 7.40835289 | 0 | C | 0 | 10 |
| 7 | 7.40845344 | T | G | 0.075 | 360 |
| 7 | 7.40848832 | 0 | G | 0 | 10 |
| 7 | 7.40853058 | 0 | C | 0 | 360 |
| 7 | 7.40858078 | 0 | G | 0 | 360 |
| 7 | 7.40889114 | T | C | 0.075 | 360 |
| 7 | 7.40909684 | G | T | 0.2 | 10 |
| 7 | 7.40926671 | A | G | 0.07263 | 358 |
| 7 | 7.40948251 | G | A | 0.075 | 360 |
| 7 | 7.40955088 | 0 | A | 0 | 10 |
| 7 | 7.40967027 | 0 | G | 0 | 360 |
| 7 | 7.40967336 | 0 | A | 0 | 360 |
| 7 | 7.40980516 | A | G | 0.002778 | 360 |
| 7 | 7.40985236 | A | G | 0.002778 | 360 |
| 7 | 7.40991392 | C | T | 0.04913 | 346 |
| 7 | 7.40991553 | T | G | 0.2213 | 348 |
| 7 | 7.40999602 | G | A | 0.04444 | 360 |
| 7 | 7.41014521 | 0 | C | 0 | 360 |
| 7 | 7.41021155 | G | A | 0.02778 | 360 |
| 7 | 7.41035462 | A | C | 0.02778 | 360 |

| | | | | | |
|---|---|---|---|---|---|
| 7 | 7.41046022 | C | T | 0.06944 | 360 |
| 7 | 7.41054208 | G | A | 0.02778 | 360 |
| 7 | 7.41075865 | A | G | 0.02778 | 360 |
| 7 | 7.41090182 | G | A | 0.07222 | 360 |
| 7 | 7.41093748 | A | G | 0.07483 | 294 |
| 7 | 7.41095494 | T | G | 0.04167 | 360 |
| 7 | 7.41097786 | 0 | C | 0 | 360 |
| 7 | 7.41102835 | C | T | 0.04167 | 360 |
| 7 | 7.41103269 | C | T | 0.07184 | 348 |
| 7 | 7.41110854 | D | I | 0.02286 | 350 |
| 7 | 7.41112601 | C | G | 0.03056 | 360 |
| 7 | 7.4111816 | 0 | G | 0 | 358 |
| 7 | 7.41118697 | T | C | 0.2771 | 350 |
| 7 | 7.41120696 | 0 | T | 0 | 360 |
| 7 | 7.41124711 | G | A | 0.07222 | 360 |
| 7 | 7.41135596 | C | T | 0.02778 | 360 |
| 7 | 7.4114705 | T | C | 0.02778 | 360 |
| 7 | 7.41152766 | C | T | 0.03056 | 360 |
| 7 | 7.41161765 | T | G | 0.02778 | 360 |
| 7 | 7.41164827 | 0 | C | 0 | 10 |
| 7 | 7.41171565 | 0 | G | 0 | 360 |
| 7 | 7.4117208 | 0 | G | 0 | 360 |
| 7 | 7.4117318 | C | T | 0.03056 | 360 |
| 7 | 7.41176104 | G | A | 0.03056 | 360 |
| 7 | 7.41176429 | 0 | G | 0 | 360 |
| 7 | 7.41181105 | G | A | 0.03056 | 360 |
| 7 | 7.41188487 | 0 | A | 0 | 326 |
| 7 | 7.41189309 | 0 | T | 0 | 360 |
| 7 | 7.41195678 | 0 | G | 0 | 312 |
| 7 | 7.41198173 | 0 | T | 0 | 360 |
| 7 | 7.41209005 | T | G | 0.02778 | 360 |
| 7 | 7.41214831 | T | G | 0.03056 | 360 |
| 7 | 7.41225703 | C | T | 0.03056 | 360 |
| 7 | 7.41240336 | 0 | G | 0 | 360 |
| 7 | 7.41248384 | G | T | 0.02778 | 360 |
| 7 | 7.41249883 | 0 | C | 0 | 358 |
| 7 | 7.41250473 | A | G | 0.02778 | 360 |
| 7 | 7.41251476 | 0 | C | 0 | 308 |
| 7 | 7.41251497 | G | C | 0.02778 | 360 |
| 7 | 7.41251782 | 0 | A | 0 | 360 |
| 7 | 7.41251956 | G | A | 0.02778 | 360 |
| 7 | 7.4125531 | G | A | 0.03073 | 358 |
| 7 | 7.41258858 | 0 | A | 0 | 360 |
| 7 | 7.41262962 | T | C | 0.02778 | 360 |
| 7 | 7.41265408 | C | A | 0.02778 | 360 |

| | | | | | |
|---|---|---|---|---|---|
| 7 | 7.41265609 | 0 | C | 0 | 360 |
| 7 | 7.41277655 | G | T | 0.05921 | 152 |
| 7 | 7.4127953 | 0 | G | 0 | 360 |
| 7 | 7.4128596 | C | T | 0.02778 | 360 |
| 7 | 7.41302561 | A | G | 0.02778 | 360 |
| 7 | 7.41304503 | 0 | C | 0 | 360 |
| 7 | 7.41306059 | G | A | 0.02778 | 360 |
| 7 | 7.41306901 | C | T | 0.1429 | 140 |
| 7 | 7.41307276 | 0 | C | 0 | 356 |
| 7 | 7.41310403 | 0 | C | 0 | 360 |
| 7 | 7.4131366 | 0 | T | 0 | 360 |
| 7 | 7.4131406 | 0 | C | 0 | 360 |
| 7 | 7.41317863 | 0 | C | 0 | 360 |
| 7 | 7.41319015 | 0 | A | 0 | 360 |
| 7 | 7.41320768 | 0 | G | 0 | 360 |
| 7 | 7.41321483 | T | C | 0.025 | 360 |
| 7 | 7.41323723 | T | C | 0.00578 | 346 |
| 7 | 7.41330248 | 0 | G | 0 | 360 |
| 7 | 7.41343215 | 0 | T | 0 | 172 |
| 7 | 7.41344259 | 0 | C | 0 | 360 |
| 7 | 7.41361705 | 0 | G | 0 | 360 |
| 7 | 7.41363583 | 0 | T | 0 | 360 |
| 7 | 7.41366102 | 0 | G | 0 | 360 |
| 7 | 7.41366744 | 0 | T | 0 | 360 |
| 7 | 7.4136692 | 0 | T | 0 | 360 |
| 7 | 7.41367037 | 0 | G | 0 | 360 |
| 7 | 7.41368577 | 0 | C | 0 | 360 |
| 7 | 7.41379561 | 0 | A | 0 | 360 |
| 7 | 7.41384516 | 0 | G | 0 | 360 |
| 7 | 7.41392649 | C | T | 0.02778 | 360 |
| 7 | 7.41403776 | 0 | G | 0 | 360 |
| 7 | 7.41411493 | 0 | A | 0 | 360 |
| 7 | 7.4141428 | 0 | T | 0 | 360 |
| 7 | 7.41418428 | 0 | C | 0 | 360 |
| 7 | 7.41420074 | 0 | G | 0 | 360 |
| 7 | 7.41421112 | 0 | G | 0 | 360 |
| 7 | 7.41425658 | 0 | C | 0 | 360 |
| 7 | 7.41442148 | 0 | G | 0 | 360 |
| 7 | 7.41454473 | A | C | 0.025 | 360 |
| 7 | 7.41464143 | 0 | C | 0 | 360 |
| 7 | 7.41470182 | A | G | 0.005556 | 360 |
| 7 | 7.41489189 | 0 | G | 0 | 360 |
| 7 | 7.41499159 | 0 | G | 0 | 360 |
| 7 | 7.41505809 | A | G | 0.025 | 360 |
| 7 | 7.4152533 | T | C | 0.025 | 360 |

| | | | | | |
|---|---|---|---|---|---|
| 7 | 7.41530954 | C | T | 0.03103 | 290 |
| 7 | 7.4153188 | 0 | G | 0 | 360 |
| 7 | 7.41540046 | A | G | 0.025 | 360 |
| 7 | 7.41540372 | 0 | T | 0 | 360 |
| 7 | 7.41549728 | C | A | 0.025 | 360 |
| 7 | 7.41550546 | T | G | 0.025 | 360 |
| 7 | 7.41562335 | C | T | 0.025 | 360 |
| 7 | 7.41580972 | 0 | T | 0 | 360 |
| 7 | 7.41583346 | 0 | G | 0 | 360 |
| 7 | 7.41587669 | T | C | 0.25 | 360 |
| 7 | 7.41588265 | 0 | G | 0 | 288 |
| 7 | 7.4158876 | 0 | A | 0 | 360 |
| 7 | 7.41590937 | 0 | A | 0 | 360 |
| 7 | 7.41591732 | 0 | G | 0 | 360 |
| 7 | 7.41596546 | 0 | C | 0 | 360 |
| 7 | 7.41611133 | A | G | 0.01944 | 360 |
| 7 | 7.41621033 | 0 | C | 0 | 352 |
| 7 | 7.41625532 | T | C | 0.01944 | 360 |
| 7 | 7.41649554 | 0 | A | 0 | 360 |
| 7 | 7.41656942 | 0 | C | 0 | 360 |
| 7 | 7.41666086 | 0 | A | 0 | 360 |
| 7 | 7.4167213 | T | G | 0.01944 | 360 |
| 7 | 7.41672282 | 0 | T | 0 | 360 |
| 7 | 7.41683917 | T | C | 0.01944 | 360 |
| 7 | 7.41686101 | G | A | 0.01944 | 360 |
| 7 | 7.41695247 | 0 | C | 0 | 360 |
| 7 | 7.41696738 | 0 | C | 0 | 352 |
| 7 | 7.41701119 | G | T | 0.2822 | 326 |
| 7 | 7.41701199 | 0 | C | 0 | 352 |
| 7 | 7.41709709 | C | T | 0.01977 | 354 |
| 7 | 7.41720401 | 0 | C | 0 | 360 |
| 7 | 7.41724558 | A | G | 0.01944 | 360 |
| 7 | 7.41734998 | 0 | C | 0 | 360 |
| 7 | 7.41745515 | C | T | 0.01944 | 360 |
| 16 | 16.44535072 | 0 | C | 0 | 360 |
| 16 | 16.44535081 | C | T | 0.09167 | 360 |
| 16 | 16.44541212 | A | C | 0.09167 | 360 |
| 16 | 16.44542416 | T | A | 0.09167 | 360 |
| 16 | 16.4455034 | T | C | 0.1408 | 348 |
| 16 | 16.44550723 | 0 | A | 0 | 360 |
| 16 | 16.44558521 | T | C | 0.4333 | 360 |
| 16 | 16.44581953 | T | C | 0.1389 | 360 |
| 16 | 16.44600931 | C | T | 0.002778 | 360 |
| 16 | 16.44607921 | 0 | C | 0 | 360 |
| 16 | 16.44619573 | A | G | 0.04444 | 360 |

| | | | | | |
|---|---|---|---|---|---|
| 16 | 16.44623632 | G | C | 0.2978 | 356 |
| 16 | 16.4463316 | G | A | 0.1389 | 360 |
| 16 | 16.44635973 | 0 | G | 0 | 360 |
| 16 | 16.44654844 | G | A | 0.2 | 10 |
| 16 | 16.44676258 | T | C | 0.04444 | 360 |
| 16 | 16.44693928 | A | G | 0.09444 | 360 |
| 16 | 16.44695743 | 0 | G | 0 | 360 |
| 16 | 16.44717054 | C | T | 0.1389 | 360 |
| 16 | 16.44736539 | 0 | G | 0 | 352 |
| 16 | 16.44737429 | 0 | T | 0 | 238 |
| 16 | 16.44750429 | T | C | 0.09167 | 360 |
| 16 | 16.44756995 | C | T | 0.1389 | 360 |
| 16 | 16.44770499 | T | C | 0.04722 | 360 |
| 16 | 16.44776914 | G | A | 0.1389 | 360 |
| 16 | 16.44786801 | C | T | 0.4361 | 360 |
| 16 | 16.44799382 | 0 | A | 0 | 360 |
| 16 | 16.44819755 | 0 | C | 0 | 360 |
| 16 | 16.44823064 | 0 | G | 0 | 360 |
| 16 | 16.44842577 | G | T | 0.3583 | 360 |
| 16 | 16.44848948 | 0 | A | 0 | 356 |
| 16 | 16.44865847 | 0 | T | 0 | 360 |
| 16 | 16.44867315 | 0 | T | 0 | 360 |
| 16 | 16.44882037 | 0 | G | 0 | 360 |
| 16 | 16.44882132 | 0 | C | 0 | 360 |
| 16 | 16.44900924 | C | T | 0.09722 | 360 |
| 16 | 16.44907944 | 0 | A | 0 | 360 |
| 16 | 16.44911965 | A | G | 0.4083 | 360 |
| 16 | 16.4491343 | 0 | G | 0 | 360 |
| 16 | 16.44932875 | 0 | G | 0 | 360 |
| 16 | 16.44937074 | A | C | 0.15 | 360 |
| 16 | 16.44948203 | A | G | 0.09722 | 360 |
| 16 | 16.44964409 | 0 | G | 0 | 360 |
| 16 | 16.4497233 | C | T | 0.4611 | 360 |
| 16 | 16.44991317 | A | G | 0.1 | 360 |
| 16 | 16.45001419 | A | G | 0.1 | 360 |
| 16 | 16.450107 | C | T | 0.425 | 360 |
| 16 | 16.45019743 | 0 | G | 0 | 354 |
| 16 | 16.4503596 | C | A | 0.1333 | 360 |
| 16 | 16.4504433 | T | C | 0.03611 | 360 |
| 16 | 16.45050214 | G | A | 0.3296 | 358 |
| 16 | 16.45053963 | G | A | 0.3306 | 360 |
| 16 | 16.45070026 | 0 | G | 0 | 348 |
| 16 | 16.45079294 | T | G | 0.3333 | 360 |
| 16 | 16.45087134 | 0 | A | 0 | 360 |
| 16 | 16.45100335 | 0 | T | 0 | 360 |

| | | | | | |
|---|---|---|---|---|---|
| 16 | 16.45110676 | A | G | 0.4667 | 360 |
| 16 | 16.45128266 | 0 | G | 0 | 360 |
| 16 | 16.45137226 | 0 | G | 0 | 360 |
| 16 | 16.4513998 | 0 | G | 0 | 360 |
| 16 | 16.45141053 | 0 | G | 0 | 360 |
| 16 | 16.45141927 | 0 | G | 0 | 360 |
| 16 | 16.45142427 | T | C | 0.4361 | 360 |
| 16 | 16.45142732 | 0 | T | 0 | 360 |
| 16 | 16.45143676 | 0 | G | 0 | 10 |
| 16 | 16.45143989 | 0 | C | 0 | 360 |
| 16 | 16.45147217 | 0 | G | 0 | 360 |
| 16 | 16.45160849 | 0 | T | 0 | 360 |
| 16 | 16.45174227 | A | C | 0.4361 | 360 |
| 16 | 16.45176755 | 0 | G | 0 | 360 |
| 16 | 16.45179953 | C | T | 0.4709 | 344 |
| 16 | 16.45188677 | A | G | 0.4361 | 360 |
| 16 | 16.45193017 | T | G | 0.4151 | 318 |
| 16 | 16.45203306 | 0 | G | 0 | 360 |
| 16 | 16.45205272 | G | A | 0.4722 | 360 |
| 16 | 16.45215021 | G | C | 0.03611 | 360 |
| 16 | 16.45227982 | A | T | 0.4361 | 360 |
| 16 | 16.45229718 | A | G | 0.03611 | 360 |
| 16 | 16.45231104 | A | C | 0.4722 | 360 |
| 16 | 16.45231819 | T | G | 0.03611 | 360 |
| 16 | 16.45232298 | T | D | 0.05263 | 190 |
| 16 | 16.45232299 | 0 | D | 0 | 170 |
| 16 | 16.45243467 | A | G | 0.4722 | 360 |
| 16 | 16.45243474 | T | C | 0.4722 | 360 |
| 16 | 16.45249298 | C | T | 0.4722 | 360 |
| 16 | 16.45261922 | C | T | 0.4722 | 360 |
| 16 | 16.45266736 | C | A | 0.4722 | 360 |
| 16 | 16.45267887 | C | T | 0.002778 | 360 |
| 16 | 16.45268418 | A | G | 0.3694 | 360 |
| 16 | 16.45269656 | A | G | 0.4722 | 360 |
| 16 | 16.45269752 | A | G | 0.3694 | 360 |
| 16 | 16.4528075 | G | A | 0.002778 | 360 |
| 16 | 16.45288581 | G | T | 0.002778 | 360 |
| 16 | 16.45297406 | 0 | C | 0 | 360 |
| 16 | 16.45316527 | 0 | C | 0 | 360 |
| 16 | 16.45317878 | A | G | 0.03611 | 360 |
| 16 | 16.45336365 | T | C | 0.025 | 360 |
| 16 | 16.45336814 | C | T | 0.03073 | 358 |
| 16 | 16.45336937 | 0 | C | 0 | 360 |
| 16 | 16.45339816 | G | A | 0.09167 | 360 |
| 16 | 16.45354917 | G | T | 0.09167 | 360 |

| | | | | | |
|---|---|---|---|---|---|
| 16 | 16.45362976 | C | A | 0.1503 | 326 |
| 16 | 16.45379507 | C | T | 0.4611 | 360 |
| 16 | 16.45389972 | T | C | 0.3722 | 360 |
| 16 | 16.45398215 | C | A | 0.4422 | 346 |
| 16 | 16.4539824 | G | T | 0.06429 | 280 |
| 16 | 16.45402199 | C | T | 0.4405 | 336 |
| 16 | 16.45406378 | 0 | T | 0 | 360 |
| 16 | 16.45410001 | T | C | 0.244 | 336 |
| 16 | 16.45416207 | A | G | 0.03889 | 360 |
| 16 | 16.45420236 | G | A | 0.03889 | 360 |
| 16 | 16.45433435 | G | A | 0.03889 | 360 |
| 16 | 16.45444656 | A | G | 0.03889 | 360 |
| 16 | 16.45450451 | G | A | 0.03889 | 360 |
| 16 | 16.45454773 | 0 | A | 0 | 360 |
| 16 | 16.45455325 | T | G | 0.03911 | 358 |
| 16 | 16.45457123 | G | A | 0.04094 | 342 |
| 16 | 16.45457191 | T | C | 0.002793 | 358 |
| 16 | 16.45469165 | A | G | 0.002778 | 360 |
| 16 | 16.45478436 | 0 | A | 0 | 360 |
| 16 | 16.45489321 | C | T | 0.03056 | 360 |
| 16 | 16.45492102 | T | C | 0.03056 | 360 |
| 16 | 16.45505642 | A | G | 0.03659 | 328 |
| 16 | 16.45521945 | 0 | C | 0 | 360 |
| 16 | 16.45522182 | G | A | 0.4694 | 360 |
| 16 | 16.45525987 | G | A | 0.002778 | 360 |
| 31 | 31.541 | 0 | T | 0 | 10 |
| 31 | 31.542 | 0 | T | 0 | 10 |
| 31 | 31.543 | 0 | C | 0 | 10 |
| 31 | 31.544 | 0 | T | 0 | 10 |
| 31 | 31.545 | 0 | T | 0 | 10 |
| 31 | 31.546 | 0 | G | 0 | 10 |
| 31 | 31.547 | 0 | T | 0 | 10 |
| 31 | 31.548 | 0 | G | 0 | 10 |
| 31 | 31.549 | 0 | T | 0 | 10 |
| 31 | 31.55 | 0 | A | 0 | 10 |
| 31 | 31.551 | 0 | C | 0 | 10 |
| 31 | 31.552 | 0 | A | 0 | 10 |
| 31 | 31.553 | 0 | A | 0 | 10 |
| 31 | 31.554 | 0 | C | 0 | 10 |
| 31 | 31.555 | 0 | G | 0 | 10 |
| 31 | 31.556 | 0 | C | 0 | 10 |
| 31 | 31.557 | 0 | C | 0 | 10 |
| 31 | 31.558 | 0 | T | 0 | 10 |
| 31 | 31.559 | 0 | A | 0 | 10 |
| 31 | 31.56 | 0 | G | 0 | 10 |

| | | | | | |
|---|---|---|---|---|---|
| 31 | 31.561 | 0 | G | 0 | 10 |
| 31 | 31.562 | 0 | A | 0 | 10 |
| 31 | 31.563 | 0 | C | 0 | 10 |
| 31 | 31.564 | 0 | T | 0 | 10 |
| 31 | 31.565 | 0 | G | 0 | 10 |
| 31 | 31.566 | 0 | G | 0 | 10 |
| 31 | 31.567 | 0 | C | 0 | 10 |
| 31 | 31.568 | 0 | A | 0 | 10 |
| 31 | 31.569 | 0 | T | 0 | 10 |
| 31 | 31.57 | 0 | T | 0 | 10 |
| 31 | 31.571 | 0 | T | 0 | 10 |
| 31 | 31.572 | 0 | T | 0 | 10 |
| 31 | 31.573 | 0 | A | 0 | 10 |
| 31 | 31.574 | 0 | C | 0 | 10 |
| 31 | 31.575 | 0 | 0 | NA | 0 |
| 31 | 31.576 | 0 | C | 0 | 10 |
| 31 | 31.577 | 0 | G | 0 | 10 |
| 31 | 31.578 | 0 | G | 0 | 10 |
| 31 | 31.579 | 0 | G | 0 | 10 |
| 31 | 31.58 | 0 | T | 0 | 10 |
| 31 | 31.581 | 0 | T | 0 | 10 |
| 31 | 31.582 | 0 | 0 | NA | 0 |
| 31 | 31.583 | 0 | A | 0 | 10 |
| 31 | 31.584 | 0 | 0 | NA | 0 |
| 31 | 31.9567 | G | T | 0.3222 | 360 |
| 31 | 31.48557 | 0 | A | 0 | 10 |
| 31 | 31.58052 | 0 | G | 0 | 360 |
| 31 | 31.65848 | 0 | G | 0 | 180 |
| 31 | 31.76431 | G | A | 0.01667 | 360 |
| 31 | 31.91801 | C | T | 0.02059 | 340 |
| 31 | 31.97289 | T | C | 0.2556 | 360 |
| 31 | 31.103502 | 0 | A | 0 | 360 |
| 31 | 31.10383 | 0 | G | 0 | 338 |
| 31 | 31.115298 | C | A | 0.01667 | 360 |
| 31 | 31.121299 | A | C | 0.01667 | 360 |
| 31 | 31.140407 | 0 | A | 0 | 360 |
| 31 | 31.176956 | 0 | T | 0 | 360 |
| 31 | 31.188664 | 0 | T | 0 | 232 |
| 31 | 31.204002 | 0 | A | 0 | 354 |
| 31 | 31.211442 | 0 | G | 0 | 360 |
| 31 | 31.230277 | T | C | 0.1917 | 360 |
| 31 | 31.242073 | T | C | 0.1917 | 360 |
| 31 | 31.244564 | C | T | 0.1917 | 360 |
| 31 | 31.273549 | T | C | 0.2709 | 358 |
| 31 | 31.273627 | 0 | T | 0 | 360 |

| | | | | | |
|---|---|---|---|---|---|
| 31 | 31.277211 | G | C | 0.1917 | 360 |
| 31 | 31.279374 | G | A | 0.1917 | 360 |
| 31 | 31.291186 | 0 | A | 0 | 360 |
| 31 | 31.306904 | C | T | 0.1917 | 360 |
| 31 | 31.314339 | 0 | A | 0 | 360 |
| 31 | 31.328034 | G | C | 0.2162 | 296 |
| 31 | 31.346846 | T | C | 0.1917 | 360 |
| 31 | 31.363505 | C | T | 0.1889 | 360 |
| 31 | 31.371302 | 0 | A | 0 | 318 |
| 31 | 31.381926 | G | C | 0.1917 | 360 |
| 31 | 31.400443 | T | G | 0.175 | 360 |
| 31 | 31.413762 | 0 | C | 0 | 360 |
| 31 | 31.426622 | A | G | 0.175 | 360 |
| 31 | 31.429557 | G | A | 0.1917 | 360 |
| 31 | 31.43871 | C | T | 0.1917 | 360 |
| 31 | 31.456608 | 0 | C | 0 | 360 |
| 31 | 31.470558 | A | G | 0.08056 | 360 |
| 31 | 31.481055 | 0 | G | 0 | 360 |
| 31 | 31.486259 | C | T | 0.175 | 360 |
| 31 | 31.491452 | C | T | 0.1917 | 360 |
| 31 | 31.497989 | A | C | 0.1917 | 360 |
| 31 | 31.508163 | T | C | 0.175 | 360 |
| 31 | 31.530468 | 0 | C | 0 | 360 |
| 31 | 31.540869 | A | G | 0.1917 | 360 |
| 31 | 31.544525 | C | A | 0.178 | 354 |
| 31 | 31.561264 | 0 | A | 0 | 352 |
| 31 | 31.577077 | T | G | 0.176 | 358 |
| 31 | 31.580882 | C | T | 0.1765 | 340 |
| 31 | 31.591784 | C | T | 0.1917 | 360 |
| 31 | 31.615158 | C | T | 0.1917 | 360 |
| 31 | 31.626586 | C | T | 0.1917 | 360 |
| 31 | 31.640075 | C | G | 0.175 | 360 |
| 31 | 31.652205 | C | T | 0.2078 | 332 |
| 31 | 31.660801 | A | G | 0.1917 | 360 |
| 31 | 31.664758 | G | A | 0.1917 | 360 |
| 31 | 31.675918 | G | A | 0.1917 | 360 |
| 31 | 31.689322 | C | A | 0.1917 | 360 |
| 31 | 31.695696 | T | C | 0.1935 | 336 |
| 31 | 31.713515 | 0 | G | 0 | 360 |
| 31 | 31.715995 | G | T | 0.1899 | 358 |
| 31 | 31.728247 | A | G | 0.175 | 360 |
| 31 | 31.739994 | T | C | 0.175 | 360 |
| 31 | 31.760993 | T | C | 0.1917 | 360 |
| 31 | 31.765196 | G | T | 0.175 | 360 |

**Table S3.** PLINK -freqx data for for MMVD candidate variant gene regions SNPs from 180 Australian CKCS.

| CHR | SNP | A1 | A2 | C(HOM A1) | C(HET) | C(HOM A2) | C(MISSING) |
|---|---|---|---|---|---|---|---|
| 2 | 2.11654206 | 0 | T | 0 | 0 | 180 | 0 |
| 2 | 2.11662236 | 0 | A | 0 | 0 | 180 | 0 |
| 2 | 2.1167411 | 0 | A | 0 | 0 | 180 | 0 |
| 2 | 2.11689791 | 0 | T | 0 | 0 | 180 | 0 |
| 2 | 2.11695641 | 0 | A | 0 | 0 | 180 | 0 |
| 2 | 2.11707593 | 0 | T | 0 | 0 | 180 | 0 |
| 2 | 2.11717132 | T | C | 0 | 7 | 173 | 0 |
| 2 | 2.11730035 | C | T | 0 | 7 | 173 | 0 |
| 2 | 2.11739358 | 0 | T | 0 | 0 | 180 | 0 |
| 2 | 2.11751842 | 0 | C | 0 | 0 | 180 | 0 |
| 2 | 2.11764533 | A | G | 0 | 7 | 173 | 0 |
| 2 | 2.11774349 | A | G | 0 | 7 | 173 | 0 |
| 2 | 2.11787735 | G | C | 1 | 11 | 168 | 0 |
| 2 | 2.11793471 | G | A | 1 | 11 | 168 | 0 |
| 2 | 2.11799338 | G | A | 1 | 11 | 168 | 0 |
| 2 | 2.118124 | 0 | G | 0 | 0 | 180 | 0 |
| 2 | 2.1182298 | A | G | 0 | 6 | 174 | 0 |
| 2 | 2.11832538 | G | A | 0 | 6 | 174 | 0 |
| 2 | 2.11843668 | 0 | A | 0 | 0 | 180 | 0 |
| 2 | 2.11855019 | 0 | C | 0 | 0 | 180 | 0 |
| 2 | 2.11871891 | A | G | 0 | 6 | 166 | 8 |
| 2 | 2.11878409 | A | G | 1 | 11 | 168 | 0 |
| 2 | 2.11900062 | 0 | G | 0 | 0 | 180 | 0 |
| 2 | 2.11901965 | 0 | T | 0 | 0 | 180 | 0 |
| 2 | 2.11914478 | 0 | C | 0 | 0 | 180 | 0 |
| 2 | 2.11924282 | A | C | 1 | 11 | 168 | 0 |
| 2 | 2.11937039 | 0 | A | 0 | 0 | 180 | 0 |
| 2 | 2.11946899 | 0 | G | 0 | 0 | 180 | 0 |
| 2 | 2.11962479 | T | G | 0 | 6 | 174 | 0 |
| 2 | 2.11979724 | G | A | 0 | 6 | 174 | 0 |
| 2 | 2.11989819 | T | C | 0 | 6 | 174 | 0 |
| 2 | 2.12002367 | C | A | 0 | 6 | 174 | 0 |
| 2 | 2.12012688 | T | C | 0 | 18 | 162 | 0 |
| 2 | 2.12019966 | C | T | 0 | 11 | 169 | 0 |
| 2 | 2.12030635 | C | T | 0 | 11 | 169 | 0 |
| 2 | 2.12039245 | 0 | A | 0 | 0 | 180 | 0 |
| 2 | 2.12053055 | 0 | T | 0 | 0 | 180 | 0 |
| 2 | 2.12063981 | C | T | 0 | 17 | 163 | 0 |
| 2 | 2.12078166 | 0 | C | 0 | 0 | 180 | 0 |
| 2 | 2.12085928 | A | G | 0 | 17 | 163 | 0 |
| 2 | 2.12102288 | 0 | C | 0 | 0 | 180 | 0 |

| | | | | | | | |
|---|---|---|---|---|---|---|---|
| 2 | 2.12108037 | C | T | 0 | 17 | 163 | 0 |
| 2 | 2.12122303 | A | G | 0 | 17 | 163 | 0 |
| 2 | 2.12126839 | T | A | 0 | 6 | 174 | 0 |
| 2 | 2.12131129 | 0 | C | 0 | 0 | 180 | 0 |
| 2 | 2.12141216 | 0 | G | 0 | 0 | 180 | 0 |
| 2 | 2.1214462 | 0 | A | 0 | 0 | 180 | 0 |
| 2 | 2.12153524 | A | G | 0 | 6 | 174 | 0 |
| 2 | 2.12162327 | 0 | C | 0 | 0 | 176 | 4 |
| 2 | 2.12174951 | C | A | 0 | 17 | 163 | 0 |
| 2 | 2.12184406 | A | G | 0 | 11 | 169 | 0 |
| 2 | 2.12192987 | G | A | 0 | 17 | 163 | 0 |
| 2 | 2.1220313 | T | G | 0 | 7 | 173 | 0 |
| 2 | 2.1221131 | C | A | 0 | 7 | 173 | 0 |
| 2 | 2.12230658 | T | C | 0 | 7 | 173 | 0 |
| 2 | 2.12234249 | A | T | 0 | 1 | 179 | 0 |
| 2 | 2.12245988 | C | T | 0 | 1 | 179 | 0 |
| 2 | 2.1225981 | T | C | 0 | 6 | 174 | 0 |
| 2 | 2.12269676 | C | A | 0 | 1 | 179 | 0 |
| 2 | 2.12283473 | A | G | 0 | 7 | 173 | 0 |
| 2 | 2.12306926 | A | G | 0 | 7 | 173 | 0 |
| 2 | 2.12318063 | G | A | 0 | 7 | 173 | 0 |
| 2 | 2.12326789 | T | C | 0 | 7 | 173 | 0 |
| 2 | 2.12347206 | G | A | 0 | 7 | 173 | 0 |
| 2 | 2.12349813 | T | C | 0 | 6 | 174 | 0 |
| 2 | 2.12361062 | C | A | 0 | 6 | 174 | 0 |
| 2 | 2.12376723 | G | T | 0 | 1 | 179 | 0 |
| 2 | 2.12386738 | A | G | 0 | 1 | 179 | 0 |
| 2 | 2.12396395 | T | A | 0 | 1 | 179 | 0 |
| 2 | 2.12413114 | T | G | 0 | 1 | 179 | 0 |
| 2 | 2.12418462 | T | G | 0 | 1 | 179 | 0 |
| 2 | 2.12434761 | 0 | A | 0 | 0 | 180 | 0 |
| 2 | 2.12442689 | 0 | G | 0 | 0 | 180 | 0 |
| 2 | 2.12453483 | T | C | 0 | 1 | 177 | 2 |
| 2 | 2.12469577 | C | T | 0 | 1 | 179 | 0 |
| 2 | 2.12478569 | A | G | 0 | 1 | 179 | 0 |
| 2 | 2.12495431 | C | T | 0 | 1 | 179 | 0 |
| 2 | 2.1250943 | 0 | A | 0 | 0 | 180 | 0 |
| 2 | 2.12512843 | T | C | 0 | 2 | 177 | 1 |
| 2 | 2.12526445 | C | T | 0 | 1 | 177 | 2 |
| 2 | 2.12533482 | C | T | 0 | 3 | 177 | 0 |
| 2 | 2.12544856 | T | C | 0 | 3 | 177 | 0 |
| 2 | 2.12565297 | C | T | 0 | 3 | 177 | 0 |
| 2 | 2.1256776 | C | T | 0 | 3 | 177 | 0 |
| 2 | 2.12585933 | T | C | 0 | 1 | 179 | 0 |
| 2 | 2.12597489 | T | C | 0 | 3 | 177 | 0 |

| | | | | | | | |
|---|---|---|---|---|---|---|---|
| 2 | 2.12611546 | G | A | 0 | 3 | 177 | 0 |
| 2 | 2.12613634 | C | T | 0 | 3 | 177 | 0 |
| 2 | 2.12624913 | 0 | A | 0 | 0 | 180 | 0 |
| 2 | 2.1264288 | 0 | T | 0 | 0 | 180 | 0 |
| 2 | 2.12643962 | C | G | 0 | 3 | 177 | 0 |
| 6 | 6.6510114 | A | G | 2 | 55 | 123 | 0 |
| 6 | 6.65112557 | T | C | 3 | 32 | 145 | 0 |
| 6 | 6.65126636 | G | A | 1 | 25 | 154 | 0 |
| 6 | 6.65136211 | G | A | 1 | 25 | 153 | 1 |
| 6 | 6.6515326 | 0 | C | 0 | 0 | 180 | 0 |
| 6 | 6.65160298 | 0 | C | 0 | 0 | 180 | 0 |
| 6 | 6.65174782 | A | G | 0 | 5 | 175 | 0 |
| 6 | 6.65180856 | T | C | 1 | 28 | 151 | 0 |
| 6 | 6.65195796 | 0 | A | 0 | 0 | 180 | 0 |
| 6 | 6.65209291 | G | A | 0 | 2 | 178 | 0 |
| 6 | 6.65221796 | G | A | 2 | 32 | 146 | 0 |
| 6 | 6.6523303 | C | A | 7 | 59 | 114 | 0 |
| 6 | 6.65246451 | C | T | 3 | 58 | 119 | 0 |
| 6 | 6.65260015 | T | C | 47 | 79 | 54 | 0 |
| 6 | 6.65270131 | T | C | 42 | 76 | 62 | 0 |
| 6 | 6.65270332 | A | G | 42 | 76 | 62 | 0 |
| 6 | 6.65276297 | T | A | 43 | 77 | 60 | 0 |
| 6 | 6.65287462 | G | T | 43 | 77 | 60 | 0 |
| 6 | 6.65298372 | A | G | 31 | 67 | 82 | 0 |
| 6 | 6.65310259 | G | A | 2 | 30 | 148 | 0 |
| 6 | 6.65319953 | G | A | 41 | 72 | 67 | 0 |
| 6 | 6.65322941 | G | A | 0 | 5 | 175 | 0 |
| 6 | 6.65332532 | T | C | 44 | 77 | 59 | 0 |
| 6 | 6.65344346 | C | T | 43 | 77 | 60 | 0 |
| 6 | 6.65348597 | A | G | 31 | 67 | 82 | 0 |
| 6 | 6.65362922 | A | G | 31 | 67 | 82 | 0 |
| 6 | 6.65386534 | A | G | 42 | 75 | 63 | 0 |
| 6 | 6.65389473 | A | G | 31 | 70 | 79 | 0 |
| 6 | 6.65406362 | C | T | 0 | 3 | 177 | 0 |
| 6 | 6.65418337 | T | G | 26 | 62 | 92 | 0 |
| 6 | 6.65423779 | A | G | 0 | 3 | 173 | 4 |
| 6 | 6.65435312 | C | T | 27 | 62 | 91 | 0 |
| 6 | 6.65447904 | T | C | 25 | 63 | 92 | 0 |
| 6 | 6.65449088 | C | T | 26 | 63 | 91 | 0 |
| 6 | 6.65463236 | T | G | 26 | 66 | 88 | 0 |
| 6 | 6.65470988 | T | C | 0 | 3 | 177 | 0 |
| 6 | 6.65480518 | 0 | G | 0 | 0 | 180 | 0 |
| 6 | 6.65496597 | T | C | 0 | 3 | 177 | 0 |
| 6 | 6.65508132 | 0 | A | 0 | 0 | 180 | 0 |
| 6 | 6.65523436 | G | A | 4 | 55 | 121 | 0 |

| | | | | | | | |
|---|---|---|---|---|---|---|---|
| 6 | 6.65525864 | A | C | 2 | 32 | 146 | 0 |
| 6 | 6.65540825 | 0 | A | 0 | 0 | 180 | 0 |
| 6 | 6.65549874 | C | T | 5 | 58 | 117 | 0 |
| 6 | 6.65567826 | 0 | C | 0 | 0 | 180 | 0 |
| 6 | 6.65578336 | A | G | 1 | 9 | 156 | 14 |
| 6 | 6.65587233 | C | T | 5 | 58 | 117 | 0 |
| 6 | 6.65595431 | 0 | G | 0 | 0 | 180 | 0 |
| 6 | 6.65596861 | C | T | 10 | 61 | 109 | 0 |
| 6 | 6.65598045 | 0 | C | 0 | 0 | 179 | 1 |
| 6 | 6.65607149 | A | G | 6 | 37 | 137 | 0 |
| 6 | 6.65618215 | T | C | 1 | 30 | 149 | 0 |
| 6 | 6.65633801 | A | C | 18 | 72 | 90 | 0 |
| 6 | 6.65645598 | 0 | A | 0 | 0 | 177 | 3 |
| 6 | 6.65656909 | T | C | 3 | 35 | 142 | 0 |
| 6 | 6.65674786 | A | G | 0 | 2 | 178 | 0 |
| 6 | 6.65675778 | C | A | 18 | 69 | 91 | 2 |
| 6 | 6.65686888 | T | C | 2 | 33 | 145 | 0 |
| 6 | 6.65705271 | C | A | 2 | 35 | 143 | 0 |
| 6 | 6.65720395 | C | A | 3 | 37 | 140 | 0 |
| 6 | 6.65723007 | G | A | 0 | 2 | 178 | 0 |
| 6 | 6.65737294 | T | C | 10 | 73 | 97 | 0 |
| 6 | 6.65746902 | G | A | 1 | 37 | 142 | 0 |
| 6 | 6.65755813 | 0 | G | 0 | 0 | 180 | 0 |
| 6 | 6.65767474 | G | A | 11 | 53 | 116 | 0 |
| 6 | 6.65781207 | A | G | 15 | 70 | 95 | 0 |
| 6 | 6.65791139 | 0 | C | 0 | 0 | 180 | 0 |
| 6 | 6.65804588 | G | A | 45 | 84 | 51 | 0 |
| 6 | 6.65822393 | A | G | 0 | 2 | 165 | 13 |
| 6 | 6.65823535 | G | A | 45 | 84 | 51 | 0 |
| 6 | 6.65823601 | G | A | 45 | 84 | 51 | 0 |
| 6 | 6.65827291 | 0 | G | 0 | 0 | 180 | 0 |
| 6 | 6.65842149 | 0 | G | 0 | 0 | 180 | 0 |
| 6 | 6.65849064 | G | T | 31 | 77 | 70 | 2 |
| 6 | 6.6586633 | G | A | 31 | 77 | 70 | 2 |
| 6 | 6.65870908 | G | T | 42 | 83 | 55 | 0 |
| 6 | 6.65882687 | A | G | 27 | 70 | 83 | 0 |
| 6 | 6.65904868 | 0 | G | 0 | 0 | 180 | 0 |
| 6 | 6.6591498 | A | G | 34 | 75 | 71 | 0 |
| 6 | 6.65917393 | T | C | 34 | 75 | 71 | 0 |
| 6 | 6.65928843 | A | G | 27 | 68 | 85 | 0 |
| 6 | 6.65928931 | C | T | 27 | 68 | 85 | 0 |
| 6 | 6.65946566 | 0 | A | 0 | 0 | 180 | 0 |
| 6 | 6.65951982 | 0 | T | 0 | 0 | 180 | 0 |
| 6 | 6.65954545 | C | T | 0 | 25 | 155 | 0 |
| 6 | 6.6596638 | 0 | C | 0 | 0 | 180 | 0 |

| | | | | | | | |
|---|---|---|---|---|---|---|---|
| 6 | 6.65975858 | A | C | 33 | 76 | 71 | 0 |
| 6 | 6.65994724 | G | A | 43 | 90 | 47 | 0 |
| 6 | 6.65998281 | G | A | 31 | 88 | 61 | 0 |
| 6 | 6.66013403 | A | G | 30 | 86 | 64 | 0 |
| 6 | 6.66024215 | C | T | 41 | 82 | 57 | 0 |
| 6 | 6.66035679 | G | A | 2 | 19 | 159 | 0 |
| 6 | 6.66048377 | T | C | 0 | 2 | 163 | 15 |
| 6 | 6.66055784 | A | G | 0 | 2 | 178 | 0 |
| 6 | 6.66070005 | 0 | C | 0 | 0 | 180 | 0 |
| 6 | 6.66088204 | 0 | G | 0 | 0 | 180 | 0 |
| 6 | 6.66098802 | 0 | G | 0 | 0 | 180 | 0 |
| 6 | 6.66106577 | C | T | 2 | 12 | 166 | 0 |
| 7 | 7.40767427 | G | T | 7 | 11 | 156 | 6 |
| 7 | 7.40778971 | C | T | 0 | 2 | 3 | 175 |
| 7 | 7.40801645 | G | A | 0 | 2 | 3 | 175 |
| 7 | 7.40812558 | T | C | 0 | 21 | 155 | 4 |
| 7 | 7.40815795 | 0 | C | 0 | 0 | 180 | 0 |
| 7 | 7.40819079 | C | G | 6 | 5 | 84 | 85 |
| 7 | 7.40823701 | T | A | 2 | 22 | 156 | 0 |
| 7 | 7.40824538 | G | A | 0 | 1 | 179 | 0 |
| 7 | 7.40827852 | 0 | A | 0 | 0 | 180 | 0 |
| 7 | 7.40835289 | 0 | C | 0 | 0 | 5 | 175 |
| 7 | 7.40845344 | T | G | 2 | 23 | 155 | 0 |
| 7 | 7.40848832 | 0 | G | 0 | 0 | 5 | 175 |
| 7 | 7.40853058 | 0 | C | 0 | 0 | 180 | 0 |
| 7 | 7.40858078 | 0 | G | 0 | 0 | 180 | 0 |
| 7 | 7.40889114 | T | C | 2 | 23 | 155 | 0 |
| 7 | 7.40909684 | G | T | 0 | 2 | 3 | 175 |
| 7 | 7.40926671 | A | G | 2 | 22 | 155 | 1 |
| 7 | 7.40948251 | G | A | 2 | 23 | 155 | 0 |
| 7 | 7.40955088 | 0 | A | 0 | 0 | 5 | 175 |
| 7 | 7.40967027 | 0 | G | 0 | 0 | 180 | 0 |
| 7 | 7.40967336 | 0 | A | 0 | 0 | 180 | 0 |
| 7 | 7.40980516 | A | G | 0 | 1 | 179 | 0 |
| 7 | 7.40985236 | A | G | 0 | 1 | 179 | 0 |
| 7 | 7.40991392 | C | T | 0 | 17 | 156 | 7 |
| 7 | 7.40991553 | T | G | 0 | 77 | 97 | 6 |
| 7 | 7.40999602 | G | A | 0 | 16 | 164 | 0 |
| 7 | 7.41014521 | 0 | C | 0 | 0 | 180 | 0 |
| 7 | 7.41021155 | G | A | 1 | 8 | 171 | 0 |
| 7 | 7.41035462 | A | C | 1 | 8 | 171 | 0 |
| 7 | 7.41046022 | C | T | 2 | 21 | 157 | 0 |
| 7 | 7.41054208 | G | A | 1 | 8 | 171 | 0 |
| 7 | 7.41075865 | A | G | 1 | 8 | 171 | 0 |
| 7 | 7.41090182 | G | A | 2 | 22 | 156 | 0 |

| | | | | | | | |
|---|---|---|---|---|---|---|---|
| 7 | 7.41093748 | A | G | 0 | 22 | 125 | 33 |
| 7 | 7.41095494 | T | G | 0 | 15 | 165 | 0 |
| 7 | 7.41097786 | 0 | C | 0 | 0 | 180 | 0 |
| 7 | 7.41102835 | C | T | 0 | 15 | 165 | 0 |
| 7 | 7.41103269 | C | T | 2 | 21 | 151 | 6 |
| 7 | 7.41110854 | D | I | 1 | 6 | 168 | 5 |
| 7 | 7.41112601 | C | G | 1 | 9 | 170 | 0 |
| 7 | 7.4111816 | 0 | G | 0 | 0 | 179 | 1 |
| 7 | 7.41118697 | T | C | 0 | 97 | 78 | 5 |
| 7 | 7.41120696 | 0 | T | 0 | 0 | 180 | 0 |
| 7 | 7.41124711 | G | A | 2 | 22 | 156 | 0 |
| 7 | 7.41135596 | C | T | 1 | 8 | 171 | 0 |
| 7 | 7.4114705 | T | C | 1 | 8 | 171 | 0 |
| 7 | 7.41152766 | C | T | 1 | 9 | 170 | 0 |
| 7 | 7.41161765 | T | G | 1 | 8 | 171 | 0 |
| 7 | 7.41164827 | 0 | C | 0 | 0 | 5 | 175 |
| 7 | 7.41171565 | 0 | G | 0 | 0 | 180 | 0 |
| 7 | 7.4117208 | 0 | G | 0 | 0 | 180 | 0 |
| 7 | 7.4117318 | C | T | 1 | 9 | 170 | 0 |
| 7 | 7.41176104 | G | A | 1 | 9 | 170 | 0 |
| 7 | 7.41176429 | 0 | G | 0 | 0 | 180 | 0 |
| 7 | 7.41181105 | G | A | 1 | 9 | 170 | 0 |
| 7 | 7.41188487 | 0 | A | 0 | 0 | 163 | 17 |
| 7 | 7.41189309 | 0 | T | 0 | 0 | 180 | 0 |
| 7 | 7.41195678 | 0 | G | 0 | 0 | 156 | 24 |
| 7 | 7.41198173 | 0 | T | 0 | 0 | 180 | 0 |
| 7 | 7.41209005 | T | G | 5 | 0 | 175 | 0 |
| 7 | 7.41214831 | T | G | 1 | 9 | 170 | 0 |
| 7 | 7.41225703 | C | T | 1 | 9 | 170 | 0 |
| 7 | 7.41240336 | 0 | G | 0 | 0 | 180 | 0 |
| 7 | 7.41248384 | G | T | 1 | 8 | 171 | 0 |
| 7 | 7.41249883 | 0 | C | 0 | 0 | 179 | 1 |
| 7 | 7.41250473 | A | G | 1 | 8 | 171 | 0 |
| 7 | 7.41251476 | 0 | C | 0 | 0 | 154 | 26 |
| 7 | 7.41251497 | G | C | 1 | 8 | 171 | 0 |
| 7 | 7.41251782 | 0 | A | 0 | 0 | 180 | 0 |
| 7 | 7.41251956 | G | A | 1 | 8 | 171 | 0 |
| 7 | 7.4125531 | G | A | 1 | 9 | 169 | 1 |
| 7 | 7.41258858 | 0 | A | 0 | 0 | 180 | 0 |
| 7 | 7.41262962 | T | C | 1 | 8 | 171 | 0 |
| 7 | 7.41265408 | C | A | 1 | 8 | 171 | 0 |
| 7 | 7.41265609 | 0 | C | 0 | 0 | 180 | 0 |
| 7 | 7.41277655 | G | T | 1 | 7 | 68 | 104 |
| 7 | 7.4127953 | 0 | G | 0 | 0 | 180 | 0 |
| 7 | 7.4128596 | C | T | 1 | 8 | 171 | 0 |

| | | | | | | | |
|---|---|---|---|---|---|---|---|
| 7 | 7.41302561 | A | G | 1 | 8 | 171 | 0 |
| 7 | 7.41304503 | 0 | C | 0 | 0 | 180 | 0 |
| 7 | 7.41306059 | G | A | 1 | 8 | 171 | 0 |
| 7 | 7.41306901 | C | T | 0 | 20 | 50 | 110 |
| 7 | 7.41307276 | 0 | C | 0 | 0 | 178 | 2 |
| 7 | 7.41310403 | 0 | C | 0 | 0 | 180 | 0 |
| 7 | 7.4131366 | 0 | T | 0 | 0 | 180 | 0 |
| 7 | 7.4131406 | 0 | C | 0 | 0 | 180 | 0 |
| 7 | 7.41317863 | 0 | C | 0 | 0 | 180 | 0 |
| 7 | 7.41319015 | 0 | A | 0 | 0 | 180 | 0 |
| 7 | 7.41320768 | 0 | G | 0 | 0 | 180 | 0 |
| 7 | 7.41321483 | T | C | 1 | 7 | 172 | 0 |
| 7 | 7.41323723 | T | C | 0 | 2 | 171 | 7 |
| 7 | 7.41330248 | 0 | G | 0 | 0 | 180 | 0 |
| 7 | 7.41343215 | 0 | T | 0 | 0 | 86 | 94 |
| 7 | 7.41344259 | 0 | C | 0 | 0 | 180 | 0 |
| 7 | 7.41361705 | 0 | G | 0 | 0 | 180 | 0 |
| 7 | 7.41363583 | 0 | T | 0 | 0 | 180 | 0 |
| 7 | 7.41366102 | 0 | G | 0 | 0 | 180 | 0 |
| 7 | 7.41366744 | 0 | T | 0 | 0 | 180 | 0 |
| 7 | 7.4136692 | 0 | T | 0 | 0 | 180 | 0 |
| 7 | 7.41367037 | 0 | G | 0 | 0 | 180 | 0 |
| 7 | 7.41368577 | 0 | C | 0 | 0 | 180 | 0 |
| 7 | 7.41379561 | 0 | A | 0 | 0 | 180 | 0 |
| 7 | 7.41384516 | 0 | G | 0 | 0 | 180 | 0 |
| 7 | 7.41392649 | C | T | 1 | 8 | 171 | 0 |
| 7 | 7.41403776 | 0 | G | 0 | 0 | 180 | 0 |
| 7 | 7.41411493 | 0 | A | 0 | 0 | 180 | 0 |
| 7 | 7.4141428 | 0 | T | 0 | 0 | 180 | 0 |
| 7 | 7.41418428 | 0 | C | 0 | 0 | 180 | 0 |
| 7 | 7.41420074 | 0 | G | 0 | 0 | 180 | 0 |
| 7 | 7.41421112 | 0 | G | 0 | 0 | 180 | 0 |
| 7 | 7.41425658 | 0 | C | 0 | 0 | 180 | 0 |
| 7 | 7.41442148 | 0 | G | 0 | 0 | 180 | 0 |
| 7 | 7.41454473 | A | C | 1 | 7 | 172 | 0 |
| 7 | 7.41464143 | 0 | C | 0 | 0 | 180 | 0 |
| 7 | 7.41470182 | A | G | 0 | 2 | 178 | 0 |
| 7 | 7.41489189 | 0 | G | 0 | 0 | 180 | 0 |
| 7 | 7.41499159 | 0 | G | 0 | 0 | 180 | 0 |
| 7 | 7.41505809 | A | G | 1 | 7 | 172 | 0 |
| 7 | 7.4152533 | T | C | 1 | 7 | 172 | 0 |
| 7 | 7.41530954 | C | T | 1 | 7 | 137 | 35 |
| 7 | 7.4153188 | 0 | G | 0 | 0 | 180 | 0 |
| 7 | 7.41540046 | A | G | 1 | 7 | 172 | 0 |
| 7 | 7.41540372 | 0 | T | 0 | 0 | 180 | 0 |

| | | | | | | | |
|---|---|---|---|---|---|---|---|
| 7 | 7.41549728 | C | A | 1 | 7 | 172 | 0 |
| 7 | 7.41550546 | T | G | 1 | 7 | 172 | 0 |
| 7 | 7.41562335 | C | T | 1 | 7 | 172 | 0 |
| 7 | 7.41580972 | 0 | T | 0 | 0 | 180 | 0 |
| 7 | 7.41583346 | 0 | G | 0 | 0 | 180 | 0 |
| 7 | 7.41587669 | T | C | 0 | 90 | 90 | 0 |
| 7 | 7.41588265 | 0 | G | 0 | 0 | 144 | 36 |
| 7 | 7.4158876 | 0 | A | 0 | 0 | 180 | 0 |
| 7 | 7.41590937 | 0 | A | 0 | 0 | 180 | 0 |
| 7 | 7.41591732 | 0 | G | 0 | 0 | 180 | 0 |
| 7 | 7.41596546 | 0 | C | 0 | 0 | 180 | 0 |
| 7 | 7.41611133 | A | G | 1 | 5 | 174 | 0 |
| 7 | 7.41621033 | 0 | C | 0 | 0 | 176 | 4 |
| 7 | 7.41625532 | T | C | 1 | 5 | 174 | 0 |
| 7 | 7.41649554 | 0 | A | 0 | 0 | 180 | 0 |
| 7 | 7.41656942 | 0 | C | 0 | 0 | 180 | 0 |
| 7 | 7.41666086 | 0 | A | 0 | 0 | 180 | 0 |
| 7 | 7.4167213 | T | G | 1 | 5 | 174 | 0 |
| 7 | 7.41672282 | 0 | T | 0 | 0 | 180 | 0 |
| 7 | 7.41683917 | T | C | 1 | 5 | 174 | 0 |
| 7 | 7.41686101 | G | A | 1 | 5 | 174 | 0 |
| 7 | 7.41695247 | 0 | C | 0 | 0 | 180 | 0 |
| 7 | 7.41696738 | 0 | C | 0 | 0 | 176 | 4 |
| 7 | 7.41701119 | G | T | 0 | 92 | 71 | 17 |
| 7 | 7.41701199 | 0 | C | 0 | 0 | 176 | 4 |
| 7 | 7.41709709 | C | T | 1 | 5 | 171 | 3 |
| 7 | 7.41720401 | 0 | C | 0 | 0 | 180 | 0 |
| 7 | 7.41724558 | A | G | 1 | 5 | 174 | 0 |
| 7 | 7.41734998 | 0 | C | 0 | 0 | 180 | 0 |
| 7 | 7.41745515 | C | T | 1 | 5 | 174 | 0 |
| 16 | 16.44535072 | 0 | C | 0 | 0 | 180 | 0 |
| 16 | 16.44535081 | C | T | 2 | 29 | 149 | 0 |
| 16 | 16.44541212 | A | C | 2 | 29 | 149 | 0 |
| 16 | 16.44542416 | T | A | 2 | 29 | 149 | 0 |
| 16 | 16.4455034 | T | C | 3 | 43 | 128 | 6 |
| 16 | 16.44550723 | 0 | A | 0 | 0 | 180 | 0 |
| 16 | 16.44558521 | T | C | 33 | 90 | 57 | 0 |
| 16 | 16.44581953 | T | C | 3 | 44 | 133 | 0 |
| 16 | 16.44600931 | C | T | 0 | 1 | 179 | 0 |
| 16 | 16.44607921 | 0 | C | 0 | 0 | 180 | 0 |
| 16 | 16.44619573 | A | G | 0 | 16 | 164 | 0 |
| 16 | 16.44623632 | G | C | 14 | 78 | 86 | 2 |
| 16 | 16.4463316 | G | A | 3 | 44 | 133 | 0 |
| 16 | 16.44635973 | 0 | G | 0 | 0 | 180 | 0 |
| 16 | 16.44654844 | G | A | 0 | 2 | 3 | 175 |

| | | | | | | | |
|---|---|---|---|---|---|---|---|
| 16 | 16.44676258 | T | C | 0 | 16 | 164 | 0 |
| 16 | 16.44693928 | A | G | 2 | 30 | 148 | 0 |
| 16 | 16.44695743 | 0 | G | 0 | 0 | 180 | 0 |
| 16 | 16.44717054 | C | T | 3 | 44 | 133 | 0 |
| 16 | 16.44736539 | 0 | G | 0 | 0 | 176 | 4 |
| 16 | 16.44737429 | 0 | T | 0 | 0 | 119 | 61 |
| 16 | 16.44750429 | T | C | 2 | 29 | 149 | 0 |
| 16 | 16.44756995 | C | T | 3 | 44 | 133 | 0 |
| 16 | 16.44770499 | T | C | 0 | 17 | 163 | 0 |
| 16 | 16.44776914 | G | A | 3 | 44 | 133 | 0 |
| 16 | 16.44786801 | C | T | 34 | 89 | 57 | 0 |
| 16 | 16.44799382 | 0 | A | 0 | 0 | 180 | 0 |
| 16 | 16.44819755 | 0 | C | 0 | 0 | 180 | 0 |
| 16 | 16.44823064 | 0 | G | 0 | 0 | 180 | 0 |
| 16 | 16.44842577 | G | T | 20 | 89 | 71 | 0 |
| 16 | 16.44848948 | 0 | A | 0 | 0 | 178 | 2 |
| 16 | 16.44865847 | 0 | T | 0 | 0 | 180 | 0 |
| 16 | 16.44867315 | 0 | T | 0 | 0 | 180 | 0 |
| 16 | 16.44882037 | 0 | G | 0 | 0 | 180 | 0 |
| 16 | 16.44882132 | 0 | C | 0 | 0 | 180 | 0 |
| 16 | 16.44900924 | C | T | 3 | 29 | 148 | 0 |
| 16 | 16.44907944 | 0 | A | 0 | 0 | 180 | 0 |
| 16 | 16.44911965 | A | G | 30 | 87 | 63 | 0 |
| 16 | 16.4491343 | 0 | G | 0 | 0 | 180 | 0 |
| 16 | 16.44932875 | 0 | G | 0 | 0 | 180 | 0 |
| 16 | 16.44937074 | A | C | 5 | 44 | 131 | 0 |
| 16 | 16.44948203 | A | G | 3 | 29 | 148 | 0 |
| 16 | 16.44964409 | 0 | G | 0 | 0 | 180 | 0 |
| 16 | 16.4497233 | C | T | 35 | 96 | 49 | 0 |
| 16 | 16.44991317 | A | G | 3 | 30 | 147 | 0 |
| 16 | 16.45001419 | A | G | 3 | 30 | 147 | 0 |
| 16 | 16.450107 | C | T | 32 | 89 | 59 | 0 |
| 16 | 16.45019743 | 0 | G | 0 | 0 | 177 | 3 |
| 16 | 16.4503596 | C | A | 3 | 42 | 135 | 0 |
| 16 | 16.4504433 | T | C | 0 | 13 | 167 | 0 |
| 16 | 16.45050214 | G | A | 17 | 84 | 78 | 1 |
| 16 | 16.45053963 | G | A | 17 | 85 | 78 | 0 |
| 16 | 16.45070026 | 0 | G | 0 | 0 | 174 | 6 |
| 16 | 16.45079294 | T | G | 18 | 84 | 78 | 0 |
| 16 | 16.45087134 | 0 | A | 0 | 0 | 180 | 0 |
| 16 | 16.45100335 | 0 | T | 0 | 0 | 180 | 0 |
| 16 | 16.45110676 | A | G | 37 | 94 | 49 | 0 |
| 16 | 16.45128266 | 0 | G | 0 | 0 | 180 | 0 |
| 16 | 16.45137226 | 0 | G | 0 | 0 | 180 | 0 |
| 16 | 16.4513998 | 0 | G | 0 | 0 | 180 | 0 |

| | | | | | | | |
|---|---|---|---|---|---|---|---|
| 16 | 16.45141053 | 0 | G | 0 | 0 | 180 | 0 |
| 16 | 16.45141927 | 0 | G | 0 | 0 | 180 | 0 |
| 16 | 16.45142427 | T | C | 33 | 91 | 56 | 0 |
| 16 | 16.45142732 | 0 | T | 0 | 0 | 180 | 0 |
| 16 | 16.45143676 | 0 | G | 0 | 0 | 5 | 175 |
| 16 | 16.45143989 | 0 | C | 0 | 0 | 180 | 0 |
| 16 | 16.45147217 | 0 | G | 0 | 0 | 180 | 0 |
| 16 | 16.45160849 | 0 | T | 0 | 0 | 180 | 0 |
| 16 | 16.45174227 | A | C | 33 | 91 | 56 | 0 |
| 16 | 16.45176755 | 0 | G | 0 | 0 | 180 | 0 |
| 16 | 16.45179953 | C | T | 37 | 88 | 47 | 8 |
| 16 | 16.45188677 | A | G | 33 | 91 | 56 | 0 |
| 16 | 16.45193017 | T | G | 18 | 96 | 45 | 21 |
| 16 | 16.45203306 | 0 | G | 0 | 0 | 180 | 0 |
| 16 | 16.45205272 | G | A | 37 | 96 | 47 | 0 |
| 16 | 16.45215021 | G | C | 0 | 13 | 167 | 0 |
| 16 | 16.45227982 | A | T | 33 | 91 | 56 | 0 |
| 16 | 16.45229718 | A | G | 0 | 13 | 167 | 0 |
| 16 | 16.45231104 | A | C | 37 | 96 | 47 | 0 |
| 16 | 16.45231819 | T | G | 0 | 13 | 167 | 0 |
| 16 | 16.45232298 | T | D | 5 | 0 | 90 | 85 |
| 16 | 16.45232299 | 0 | D | 0 | 0 | 85 | 95 |
| 16 | 16.45243467 | A | G | 37 | 96 | 47 | 0 |
| 16 | 16.45243474 | T | C | 37 | 96 | 47 | 0 |
| 16 | 16.45249298 | C | T | 37 | 96 | 47 | 0 |
| 16 | 16.45261922 | C | T | 37 | 96 | 47 | 0 |
| 16 | 16.45266736 | C | A | 37 | 96 | 47 | 0 |
| 16 | 16.45267887 | C | T | 0 | 1 | 179 | 0 |
| 16 | 16.45268418 | A | G | 22 | 89 | 69 | 0 |
| 16 | 16.45269656 | A | G | 37 | 96 | 47 | 0 |
| 16 | 16.45269752 | A | G | 22 | 89 | 69 | 0 |
| 16 | 16.4528075 | G | A | 0 | 1 | 179 | 0 |
| 16 | 16.45288581 | G | T | 0 | 1 | 179 | 0 |
| 16 | 16.45297406 | 0 | C | 0 | 0 | 180 | 0 |
| 16 | 16.45316527 | 0 | C | 0 | 0 | 180 | 0 |
| 16 | 16.45317878 | A | G | 0 | 13 | 167 | 0 |
| 16 | 16.45336365 | T | C | 4 | 1 | 175 | 0 |
| 16 | 16.45336814 | C | T | 0 | 11 | 168 | 1 |
| 16 | 16.45336937 | 0 | C | 0 | 0 | 180 | 0 |
| 16 | 16.45339816 | G | A | 2 | 29 | 149 | 0 |
| 16 | 16.45354917 | G | T | 2 | 29 | 149 | 0 |
| 16 | 16.45362976 | C | A | 2 | 45 | 116 | 17 |
| 16 | 16.45379507 | C | T | 36 | 94 | 50 | 0 |
| 16 | 16.45389972 | T | C | 23 | 88 | 69 | 0 |
| 16 | 16.45398215 | C | A | 32 | 89 | 52 | 7 |

| | | | | | | | |
|---|---|---|---|---|---|---|---|
| 16 | 16.4539824 | G | T | 0 | 18 | 122 | 40 |
| 16 | 16.45402199 | C | T | 29 | 90 | 49 | 12 |
| 16 | 16.45406378 | 0 | T | 0 | 0 | 180 | 0 |
| 16 | 16.45410001 | T | C | 0 | 82 | 86 | 12 |
| 16 | 16.45416207 | A | G | 0 | 14 | 166 | 0 |
| 16 | 16.45420236 | G | A | 0 | 14 | 166 | 0 |
| 16 | 16.45433435 | G | A | 0 | 14 | 166 | 0 |
| 16 | 16.45444656 | A | G | 0 | 14 | 166 | 0 |
| 16 | 16.45450451 | G | A | 0 | 14 | 166 | 0 |
| 16 | 16.45454773 | 0 | A | 0 | 0 | 180 | 0 |
| 16 | 16.45455325 | T | G | 0 | 14 | 165 | 1 |
| 16 | 16.45457123 | G | A | 0 | 14 | 157 | 9 |
| 16 | 16.45457191 | T | C | 0 | 1 | 178 | 1 |
| 16 | 16.45469165 | A | G | 0 | 1 | 179 | 0 |
| 16 | 16.45478436 | 0 | A | 0 | 0 | 180 | 0 |
| 16 | 16.45489321 | C | T | 0 | 11 | 169 | 0 |
| 16 | 16.45492102 | T | C | 0 | 11 | 169 | 0 |
| 16 | 16.45505642 | A | G | 0 | 12 | 152 | 16 |
| 16 | 16.45521945 | 0 | C | 0 | 0 | 180 | 0 |
| 16 | 16.45522182 | G | A | 37 | 95 | 48 | 0 |
| 16 | 16.45525987 | G | A | 0 | 1 | 179 | 0 |
| 31 | 31.541 | 0 | T | 0 | 0 | 5 | 175 |
| 31 | 31.542 | 0 | T | 0 | 0 | 5 | 175 |
| 31 | 31.543 | 0 | C | 0 | 0 | 5 | 175 |
| 31 | 31.544 | 0 | T | 0 | 0 | 5 | 175 |
| 31 | 31.545 | 0 | T | 0 | 0 | 5 | 175 |
| 31 | 31.546 | 0 | G | 0 | 0 | 5 | 175 |
| 31 | 31.547 | 0 | T | 0 | 0 | 5 | 175 |
| 31 | 31.548 | 0 | G | 0 | 0 | 5 | 175 |
| 31 | 31.549 | 0 | T | 0 | 0 | 5 | 175 |
| 31 | 31.55 | 0 | A | 0 | 0 | 5 | 175 |
| 31 | 31.551 | 0 | C | 0 | 0 | 5 | 175 |
| 31 | 31.552 | 0 | A | 0 | 0 | 5 | 175 |
| 31 | 31.553 | 0 | A | 0 | 0 | 5 | 175 |
| 31 | 31.554 | 0 | C | 0 | 0 | 5 | 175 |
| 31 | 31.555 | 0 | G | 0 | 0 | 5 | 175 |
| 31 | 31.556 | 0 | C | 0 | 0 | 5 | 175 |
| 31 | 31.557 | 0 | C | 0 | 0 | 5 | 175 |
| 31 | 31.558 | 0 | T | 0 | 0 | 5 | 175 |
| 31 | 31.559 | 0 | A | 0 | 0 | 5 | 175 |
| 31 | 31.56 | 0 | G | 0 | 0 | 5 | 175 |
| 31 | 31.561 | 0 | G | 0 | 0 | 5 | 175 |
| 31 | 31.562 | 0 | A | 0 | 0 | 5 | 175 |
| 31 | 31.563 | 0 | C | 0 | 0 | 5 | 175 |
| 31 | 31.564 | 0 | T | 0 | 0 | 5 | 175 |

| | | | | | | | |
|---|---|---|---|---|---|---|---|
| 31 | 31.565 | 0 | G | 0 | 0 | 5 | 175 |
| 31 | 31.566 | 0 | G | 0 | 0 | 5 | 175 |
| 31 | 31.567 | 0 | C | 0 | 0 | 5 | 175 |
| 31 | 31.568 | 0 | A | 0 | 0 | 5 | 175 |
| 31 | 31.569 | 0 | T | 0 | 0 | 5 | 175 |
| 31 | 31.57 | 0 | T | 0 | 0 | 5 | 175 |
| 31 | 31.571 | 0 | T | 0 | 0 | 5 | 175 |
| 31 | 31.572 | 0 | T | 0 | 0 | 5 | 175 |
| 31 | 31.573 | 0 | A | 0 | 0 | 5 | 175 |
| 31 | 31.574 | 0 | C | 0 | 0 | 5 | 175 |
| 31 | 31.575 | 0 | 0 | 0 | 0 | 0 | 180 |
| 31 | 31.576 | 0 | C | 0 | 0 | 5 | 175 |
| 31 | 31.577 | 0 | G | 0 | 0 | 5 | 175 |
| 31 | 31.578 | 0 | G | 0 | 0 | 5 | 175 |
| 31 | 31.579 | 0 | G | 0 | 0 | 5 | 175 |
| 31 | 31.58 | 0 | T | 0 | 0 | 5 | 175 |
| 31 | 31.581 | 0 | T | 0 | 0 | 5 | 175 |
| 31 | 31.582 | 0 | 0 | 0 | 0 | 0 | 180 |
| 31 | 31.583 | 0 | A | 0 | 0 | 5 | 175 |
| 31 | 31.584 | 0 | 0 | 0 | 0 | 0 | 180 |
| 31 | 31.9567 | G | T | 19 | 78 | 83 | 0 |
| 31 | 31.48557 | 0 | A | 0 | 0 | 5 | 175 |
| 31 | 31.58052 | 0 | G | 0 | 0 | 180 | 0 |
| 31 | 31.65848 | 0 | G | 0 | 0 | 90 | 90 |
| 31 | 31.76431 | G | A | 0 | 6 | 174 | 0 |
| 31 | 31.91801 | C | T | 0 | 7 | 163 | 10 |
| 31 | 31.97289 | T | C | 9 | 74 | 97 | 0 |
| 31 | 31.103502 | 0 | A | 0 | 0 | 180 | 0 |
| 31 | 31.10383 | 0 | G | 0 | 0 | 169 | 11 |
| 31 | 31.115298 | C | A | 0 | 6 | 174 | 0 |
| 31 | 31.121299 | A | C | 0 | 6 | 174 | 0 |
| 31 | 31.140407 | 0 | A | 0 | 0 | 180 | 0 |
| 31 | 31.176956 | 0 | T | 0 | 0 | 180 | 0 |
| 31 | 31.188664 | 0 | T | 0 | 0 | 116 | 64 |
| 31 | 31.204002 | 0 | A | 0 | 0 | 177 | 3 |
| 31 | 31.211442 | 0 | G | 0 | 0 | 180 | 0 |
| 31 | 31.230277 | T | C | 7 | 55 | 118 | 0 |
| 31 | 31.242073 | T | C | 7 | 55 | 118 | 0 |
| 31 | 31.244564 | C | T | 7 | 55 | 118 | 0 |
| 31 | 31.273549 | T | C | 12 | 73 | 94 | 1 |
| 31 | 31.273627 | 0 | T | 0 | 0 | 180 | 0 |
| 31 | 31.277211 | G | C | 7 | 55 | 118 | 0 |
| 31 | 31.279374 | G | A | 7 | 55 | 118 | 0 |
| 31 | 31.291186 | 0 | A | 0 | 0 | 180 | 0 |
| 31 | 31.306904 | C | T | 7 | 55 | 118 | 0 |

| | | | | | | | |
|---|---|---|---|---|---|---|---|
| 31 | 31.314339 | 0 | A | 0 | 0 | 180 | 0 |
| 31 | 31.328034 | G | C | 13 | 38 | 97 | 32 |
| 31 | 31.346846 | T | C | 7 | 55 | 118 | 0 |
| 31 | 31.363505 | C | T | 7 | 54 | 119 | 0 |
| 31 | 31.371302 | 0 | A | 0 | 0 | 159 | 21 |
| 31 | 31.381926 | G | C | 7 | 55 | 118 | 0 |
| 31 | 31.400443 | T | G | 5 | 53 | 122 | 0 |
| 31 | 31.413762 | 0 | C | 0 | 0 | 180 | 0 |
| 31 | 31.426622 | A | G | 5 | 53 | 122 | 0 |
| 31 | 31.429557 | G | A | 7 | 55 | 118 | 0 |
| 31 | 31.43871 | C | T | 7 | 55 | 118 | 0 |
| 31 | 31.456608 | 0 | C | 0 | 0 | 180 | 0 |
| 31 | 31.470558 | A | G | 1 | 27 | 152 | 0 |
| 31 | 31.481055 | 0 | G | 0 | 0 | 180 | 0 |
| 31 | 31.486259 | C | T | 5 | 53 | 122 | 0 |
| 31 | 31.491452 | C | T | 7 | 55 | 118 | 0 |
| 31 | 31.497989 | A | C | 7 | 55 | 118 | 0 |
| 31 | 31.508163 | T | C | 5 | 53 | 122 | 0 |
| 31 | 31.530468 | 0 | C | 0 | 0 | 180 | 0 |
| 31 | 31.540869 | A | G | 7 | 55 | 118 | 0 |
| 31 | 31.544525 | C | A | 5 | 53 | 119 | 3 |
| 31 | 31.561264 | 0 | A | 0 | 0 | 176 | 4 |
| 31 | 31.577077 | T | G | 5 | 53 | 121 | 1 |
| 31 | 31.580882 | C | T | 7 | 46 | 117 | 10 |
| 31 | 31.591784 | C | T | 7 | 55 | 118 | 0 |
| 31 | 31.615158 | C | T | 7 | 55 | 118 | 0 |
| 31 | 31.626586 | C | T | 7 | 55 | 118 | 0 |
| 31 | 31.640075 | C | G | 5 | 53 | 122 | 0 |
| 31 | 31.652205 | C | T | 7 | 55 | 104 | 14 |
| 31 | 31.660801 | A | G | 7 | 55 | 118 | 0 |
| 31 | 31.664758 | G | A | 7 | 55 | 118 | 0 |
| 31 | 31.675918 | G | A | 7 | 55 | 118 | 0 |
| 31 | 31.689322 | C | A | 7 | 55 | 118 | 0 |
| 31 | 31.695696 | T | C | 5 | 55 | 108 | 12 |
| 31 | 31.713515 | 0 | G | 0 | 0 | 180 | 0 |
| 31 | 31.715995 | G | T | 7 | 54 | 118 | 1 |
| 31 | 31.728247 | A | G | 5 | 53 | 122 | 0 |
| 31 | 31.739994 | T | C | 5 | 53 | 122 | 0 |
| 31 | 31.760993 | T | C | 7 | 55 | 118 | 0 |
| 31 | 31.765196 | G | T | 5 | 53 | 122 | 0 |
